# Patterns and determinants of antenatal care utilization: analysis of national survey data in seven countdown countries

**DOI:** 10.7189/jogh.06.010404

**Published:** 2016-06

**Authors:** Ghada Saad–Haddad, Jocelyn DeJong, Nancy Terreri, María Clara Restrepo–Méndez, Jamie Perin, Lara Vaz, Holly Newby, Agbessi Amouzou, Aluísio JD Barros, Jennifer Bryce

**Affiliations:** 1 Faculty of Health Sciences, American University of Beirut, Beirut, Lebanon; 2 Independent consultant; 3 Federal University of Pelotas, Pelotas, Brazil; 4 Institute for International Programs, Johns Hopkins Bloomberg School of Public Health, Baltimore, MD, USA; 5 Save the Children, Washington D.C., USA; 6 Division of Data, Research and Policy, United Nations Children’s Fund, New York City, NY, USA; 7 Institute for International Programs, Johns Hopkins Bloomberg School of Public Health, Baltimore, MD, USA

## Abstract

**Background:**

Antenatal care (ANC) is critical for improving maternal and newborn health. WHO recommends that pregnant women complete at least four ANC visits. Countdown and other global monitoring efforts track the proportions of women who receive one or more visits by a skilled provider (ANC1+) and four or more visits by any provider (ANC4+). This study investigates patterns of drop–off in use between ANC1+ and ANC4+, and explores inequalities in women’s use of ANC services. It also identifies determinants of utilization and describes countries’ ANC–related policies, and programs.

**Methods:**

We performed secondary analyses using Demographic Health Survey (DHS) data from seven Countdown countries: Bangladesh, Cambodia, Cameroon, Nepal, Peru, Senegal and Uganda. The descriptive analysis illustrates country variations in the frequency of visits by provider type, content, and by household wealth, women’s education and type of residence. We conducted a multivariable analysis using a conceptual framework to identify determinants of ANC utilization. We collected contextual information from countries through a standard questionnaire completed by country–based informants.

**Results:**

Each country had a unique pattern of ANC utilization in terms of coverage, inequality and the extent to which predictors affected the frequency of visits. Nevertheless, common patterns arise. Women having four or more visits usually saw a skilled provider at least once, and received more evidence–based content interventions than women reporting fewer than four visits. A considerable proportion of women reporting four or more visits did not report receiving the essential interventions. Large disparities exist in ANC use by household wealth, women’s education and residence area; and are wider for a larger number of visits. The multivariable analyses of two models in each country showed that determinants had different effects on the dependent variable in each model. Overall, strong predictors of ANC initiation and having a higher frequency (4+) of visits were woman’s education and household wealth. Gestational age at first visit, birth rank and preceding birth interval were generally negatively associated with initiating visits and with having four or more visits. Information on country policies and programs were somewhat informative in understanding the utilization patterns across the countries, although timing of adoption and actual implementation make direct linkages impossible to verify.

**Conclusion:**

Secondary analyses provided a more detailed picture of ANC utilization patterns in the seven countries. While coverage levels differ by country and sub–groups, all countries can benefit from specific in–country assessments to properly identify the underserved women and the reasons behind low coverage and missed interventions. Overall, emphasis needs to be put on assessing the quality of care offered and identifying women’s perception to the care as well as the barriers hindering utilization. Country policies and programs need to be reviewed, evaluated and/or implemented properly to ensure that women receive the recommended number of ANC visits with appropriate content, especially, poor and less educated women residing in rural areas.

Antenatal care (ANC), defined as the care provided to a woman during her pregnancy, is an essential component of reproductive health care. ANC can serve as a platform for the delivery of highly–effective health interventions that can reduce preventable maternal and newborn deaths [1,2]. ANC services offer pregnant women an entry point to the health care system, providing appropriate screening, intervention and treatment throughout pregnancy, and encouraging women to seek a skilled birth attendant for their delivery [3]. Furthermore, using ANC allows women to receive information about improving maternal health through proper nutrition and self–care during pregnancy; and throughout the postpartum period, such as the benefits of exclusive breastfeeding and counseling on family planning methods [4].

The current World Health Organization (WHO) recommendation is that each woman receives a minimum of four goal–oriented or focused ANC visits for low–risk deliveries, to be supervised or attended by a skilled ANC attendant [4]. The timing of the first visit should be before 16 weeks of pregnancy, the second visit should be between 24 and 26 weeks, the third visit between 30 and 32 weeks, and the fourth visit between 36 and 38 weeks [5]. WHO defines a thorough set of essential elements for each visit (**Box 1**).

Box 1 **The evolution of World Health Organization guidelines for antenatal care visits**

The concept of antenatal care originated in Europe in the early decades of the 20^th^ century. It is believed that the ANC model and the recommendations set at that time formed the foundation for ANC programs worldwide. The model indicated that visits should begin around 16 weeks of gestation, followed by visits at 24 and 28 weeks, then fortnightly visits until 36 weeks, and finally, weekly visits until delivery [6].

This ‘Western model’ was implemented for developing countries without taking into consideration contextual factors, which are especially important in low–resource settings [7]. WHO therefore developed a new model of ANC, consisting of a reduced number of visits and specifying the evidence–based interventions to be provided at each visit, including: assessment of the pregnant woman; screening for pre–eclampsia, anemia, syphilis, and HIV; provision of preventive measures such as checking of iron and folate dosage, tetanus toxoid immunization, anti–malarial precautions, and advice on labor or danger signs; advise on proper self–care, nutrition, and substance abuse; and counseling on the importance of family planning [5]. These recommendations are referred to as “focused” or “goal–oriented” ANC. Clinical evidence at the time the recommendations were released indicated that health outcomes were similar for women who received the four focused visits and women who received standard ANC with more visits [7,8].

Dowswell and colleagues [6] in an updated Cochrane systematic review using new methods of assessment, showed a statistically significant increase in perinatal mortality in low– and middle–income settings among women who received focused ANC compared to women who received standard ANC. In a 2011 statement, WHO acknowledged this and planned to provide updated guidelines for ANC based on their findings to be generated from additional secondary analyses [9]. The results of a secondary analysis looking at the WHO ANC trial were published in 2013, again showing a substantial increase in perinatal mortality among women receiving the focused ANC compared to those receiving the standard package, especially between 32 and 36 weeks of gestation. However, the findings also showed high levels of heterogeneity between the populations in the trials, and suggested that differences in perinatal mortality between the control and intervention groups could be attributed to different settings, populations or even quality of care received [10]. The WHO is re–evaluating its ANC guidelines, an exercise which is expected to be completed by the end of 2015 [11].

Coverage of ANC has been used globally as one of the indicators to track progress towards Target 5.B (achieving universal access to reproductive health by 2015) under Millennium Development Goal 5 (MDG 5) to improve maternal health [12]. The official ANC indicators for global tracking are: (1) the proportion of women with a recent live birth who report at least one ANC visit with skilled health personnel (ANC 1+); and (2) the proportion of women with a recent live birth who report at least four ANC visits with any provider (ANC 4+) [12].The Countdown to 2015 for Maternal, Newborn and Child Survival, a global movement that tracks coverage for evidence–based interventions in 75 countries that account for more than 95% of maternal and child deaths [13], also reports on the ANC 1+ and ANC 4+ indicators.

There have been numerous studies of the determinants of ANC use in low– and middle–income countries. Fewer studies have examined the determinants of use by frequency of antenatal care visits, comparatively, and through inferential analyses [14-21]. There have also been several analyses of equity in utilization of ANC services. Relevant articles stratify utilization by urban/rural place of residence [16,22–24], and less frequently, by mother’s education [15,25], wealth [15,26], income [25], and ethnicity [25]. However, little is known about the frequency of ANC visits in general, especially as a comparative presentation across countries. No previous study, to our knowledge, has examined utilization in terms of what the globally measured ANC indicators might be missing with respect to associations between women’s characteristics and their patterns of visits. Moreover, qualitative studies, or studies that use both qualitative and quantitative methods, are fewer in number [27]. These studies focus on contextual aspects such as the presence of health care workers in the community, availability of affordable care, household characteristics and perceived distance from the health care facility, waiting time at the facility [27], women’s perceptions about ANC, and their experiences, attitudes, beliefs and perceived need for services [27,28].

For this paper, we purposely selected a limited number of Countdown countries to examine and understand the underlying patterns of ANC utilization that are not revealed when relying solely on the globally measured ANC indicators. We identify whether a significant drop–off in utilization occurs after a certain number of visits. We also describe the number of ANC visits by the type of provider, and the content received overall during ANC. We examine the coverage of ANC by three measures of inequality. Finally, we use several environmental, population and individual characteristics to analyze utilization patterns in the selected countries. In addition, contextual information on policy and program structure was collected from the selected countries for the purpose of improving understanding of ANC coverage levels and drop off.

## DATA AND METHODOLOGY

### Data

The selection of countries was based on several criteria with the desire to have six to seven Countdown countries from different world regions, each with a Demographic Health Survey (DHS) in 2010 or later. We chose countries with extreme coverage levels (high or low) of ANC 4+, ANC 1+ and skilled birth attendance. We also selected a couple of countries identified as priority countries for eliminating mother–to–child transmission of HIV (list of the Countdown countries in Table S1 in **Online Supplementary Document(Online Supplementary Document)**). The seven selected Countdown countries are Bangladesh, Cambodia, Cameroon, Nepal, Peru, Senegal and Uganda.

Data for our analyses were obtained from nationally–representative household surveys conducted under the DHS program [29]. Information on the number of ANC visits for the most recent live birth in the five years preceding the survey for each woman in the sample was found in the women’s individual questionnaire. Women who responded “don’t know” or had a missing response were excluded from the analysis. Missing variables found in responses to the other variables we chose for our descriptive and inferential statistics were handled similarly and observations were dropped from the analysis (proportions of missing varied between variables in each country and across countries but never exceed a proportion of 0.7% of the total sample size per country). The following DHS surveys were used for the analyses presented in this work: Bangladesh 2011, Cambodia 2010, Cameroon 2011, Nepal 2011, Peru 2012, Senegal 2010, Uganda 2011.

### Methodology

The bulk of this study consisted of a thorough descriptive analysis to unpack the ANC indicators. We analyzed ANC visit frequency by type of provider reported and by content interventions received. In each survey, women were asked to list the providers they saw during any ANC visit. We categorized women who reported having ANC into those who saw a skilled provider for at least one visit and those who saw unskilled providers only (the list of providers in Table S2 in **Online Supplementary Document(Online Supplementary Document)**). We selected a limited number of evidence–based content interventions that should be routinely administered during ANC visits and data usually available through DHS: blood sample taken, blood pressure taken, urine sample taken and being told about pregnancy problems. In Bangladesh, only being told about pregnancy problems was available, as the other questions were not asked. We examined differences in content received among women who saw a skilled provider vs those who saw only an unskilled provider, and the pattern of content received by wealth quintile. We charted the frequency of visits by gestational age at first ANC visit (by trimester). In Bangladesh, no data were collected on this variable. We also investigated inequalities in utilization of ANC visits by three dimensions of inequality, specifically, women’s education (none, primary, secondary, and higher), household wealth quintile (five wealth quintiles from poorest to richest as defined by DHS) and the area of residence (rural or urban). We described the differences in proportions of women’s reported frequency of visits by each of the three dimensions.

**Figure 1:**
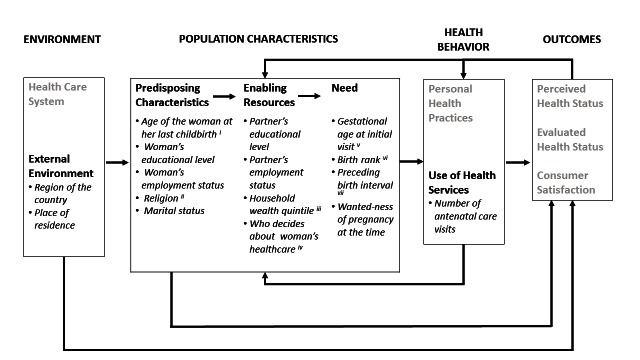
The conceptual framework based on Anderson’s Behavioral Model of Healthcare Use and the corresponding determinants used in our secondary analysis [31]. Source: Anderson 1995 [31]. ^i^ Age at woman’s most recent birth was calculated by subtracting the last child’s date of birth from the woman’s date of birth divided by 12. ^ii^Religion was categorized as dominant religion and other religions. ^iii^Household wealth quintile is made up of five wealth quintiles from poorest to richest as constructed by DHS where each quintile represents 20% of the households in the study sample. ^iv^The variable ‘who decides about woman’s health care’ is categorized as: woman alone, woman & partner, partner alone, someone else. ^v^Gestational age at first ANC visit was grouped into trimesters. ^vi^Birth rank was categorized as: 1^st^ – 2^nd^ birth, 3^rd^ – 4^th^ birth, 5^th^ birth or more. ^vii^Preceding birth interval was grouped into: first birth (no interval), less than 2 years interval, 2–3 years interval, more than 3 years interval.

To identify the determinants that affected women’s choices in initiating ANC and the seeking patterns among women who reported receiving ANC, we adopted Anderson’s Behavioral Model for Healthcare Use [30], specifically four components of the model and a selection of 15 determinants (**Figure 1**). Anderson’s Behavioral Model has been used extensively to understand utilization in different health care settings [32]. Numerous studies have made use of this conceptual model to study the determinants of antenatal care utilization [17,19,32,33]. We assessed the factors that influence the frequency of ANC visits for two comparisons: those reporting no visits vs those reporting one or more visits; and those reporting one to three visits vs those reporting four or more visits. After examining the bivariate relationships between each determinant and the dependent outcomes, we performed multivariable logistic regression analyses. Using the strategy of hierarchical entry of variables [34], we first included the external environment factors into the models to assess their association with the outcome variable. Using backward elimination we exclude factors not significant (*P* < 0.05) at the level being entered, one at a time, starting with the variable with the highest *P*–value. We re–ran the models until all the variables at the level being entered were significant. After looking at the model with environmental variables only, we added the predisposing characteristics to the models followed by a reapplication of the backward elimination procedure. The enabling factors and the need factors were then added to the models using similar procedures.

We analyzed the data with Stata (StataCorp. 2013. Stata Statistical Software: Release 13; College Station, TX: StataCorp LP) using the ‘svy’ prefix to take into account the complex sample design, including sampling weights and clustering.

### Collection of information on ANC–related national policies and programs

We collected information on policy and programming for ANC in each of the seven countries by identifying a government official, researcher and/or non–governmental organization (NGO) staff knowledgeable about current and past ANC policy and programs and willing to assist. Each key informant was encouraged to contact additional resource persons as necessary and to provide the research team with copies of relevant documents. We developed and provided informants with a self–administered standard. Topics covered in the questionnaire included information on country policies and guidelines, with details on recommended timing, number and content of ANC visits. Additional information requested included the locations of ANC service provision in each country, the types of providers, incentives for women to seek care, user fees, incentives for providers, communication or social marketing around ANC, and how the ANC service is organized. Most questions were open–ended, and a final question asked for any additional comments from the informant on how uptake of ANC services might be improved. To assist the informant in filling out the questionnaire and for verification purposes, each country questionnaire included pre–completed descriptive information from the latest country DHS or other publication, when available.

One member of the research team reviewed global policies relevant to ANC, compiled results from the country questionnaires, reviewed documents provided by key informants and others found through online searches. Follow–up with key informants was made to provide missing information or to resolve discrepancies. Descriptive summaries and tables were completed for each country along with an overall summary of findings. Results were shared with the research team and used in analyzing the country results.

## RESULTS

The seven countries we selected for this secondary analysis are Bangladesh, Cambodia, Cameroon, Nepal, Peru, Senegal and Uganda. The un–weighted sample sizes of women aged 15–49 years in each country were: 7319 (ever–married women only) in Bangladesh; 6421 women in Cambodia; 7576 women in Cameroon; 4079 women in Nepal; 7991 women in Peru; 8008 women in Senegal and 4818 women in Uganda.

### Descriptive analysis

**Table 1** describes the proportions of women reporting one or more and four or more ANC visits with a skilled provider or any provider. With the exception of Bangladesh and Nepal, 85–96% of the women reported at least one ANC visit with a skilled provider. In Bangladesh, around one–third of the women reported not receiving any ANC. The proportion of women who reported four or more visits ranged from 48% to 63% in five of the seven countries. Bangladesh was at the low end, with 24% and Peru stood out with 94%. Despite the fact that the globally measured ANC indicators are not fully comparable, because the ANC 1+ indicator refers to visits with a skilled provider and ANC 4+ refers to visits with any provider, it is important to note that in five out of the seven countries, over 90% of the women who reported receiving four or more visits with any provider also reported receiving at least one visit with a skilled provider (**Table 1**).

**Table 1:** Percentage of women who had a live birth in the five years preceding the DHS surveys who reported one ANC visit with a skilled provider and four or more visits with any provider or skilled provider for their most recent live birth, in seven Countdown countries

	One or more ANC visits with any provider (%)	ANC 1+ (with a skilled provider) (%)	ANC 4+ (with any provider) (%)	Four or more ANC visits with a skilled provider* (%)	Women reporting ANC 4+ with any provider and present as a subset among women reporting ANC 1+ with a skilled provider (%)
**Bangladesh 2011**	64.6	51.7	23.9	19.9	83.4
**Cambodia 2010**	89.6	89.1	59.6	59.4	99.7
**Cameroon 2011**	85.4	84.9	62.9	62.7	99.7
**Nepal 2011**	84.9	58.2	50.1	40.0	79.8
**Peru 2012**	98.4	96.0	94.4	92.2	97.6
**Senegal 2010**	95.8	93.2	51.2	50.2	98.1
**Uganda 2011**	95.7	94.8	48.5	48.1	99.3

DHS – Demographic Health survey, ANC – antenatal care

*At least one visit of the four or more visits is with a skilled provider.

**Figure 2:**
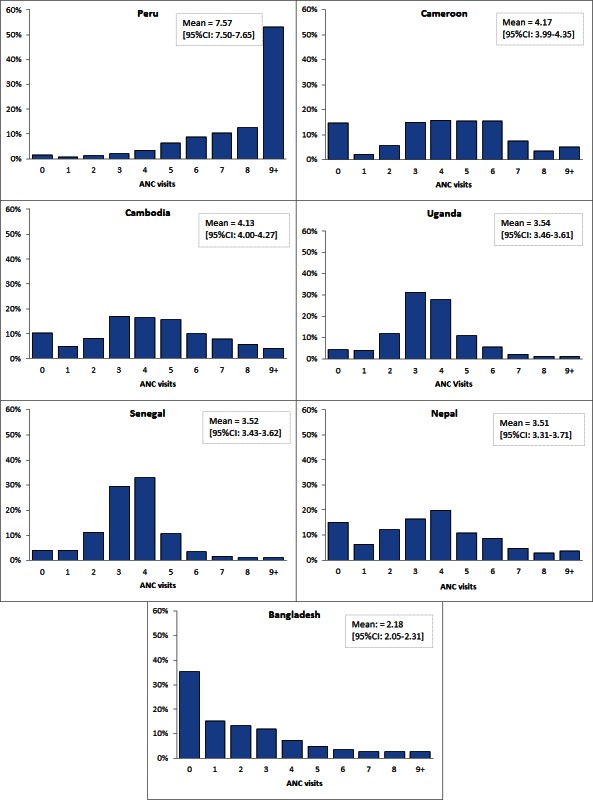
Percentage of women who had a live birth in the five years preceding the DHS surveys reporting zero to more than nine ANC visits for their most recent live birth, and mean of ANC visits among all these women (95% confidence intervals), in seven Countdown countries.

The distribution of number of ANC visits varies from country to country, as shown in **Figure 2**. Peru has the highest mean (7.6); Cameroon and Cambodia have a mean of just over four reported ANC visits. The distributions in Bangladesh and Peru represent two extremes, with a right skewed distribution in Bangladesh (35% of women with no visits) and a left skewed distribution in Peru (nearly no women reporting zero ANC visits). Most women who reported no ANC visits reside in rural areas, are in the two poorest quintiles of their national populations, and have less than a primary school education (data not shown).

**Figure 3:**
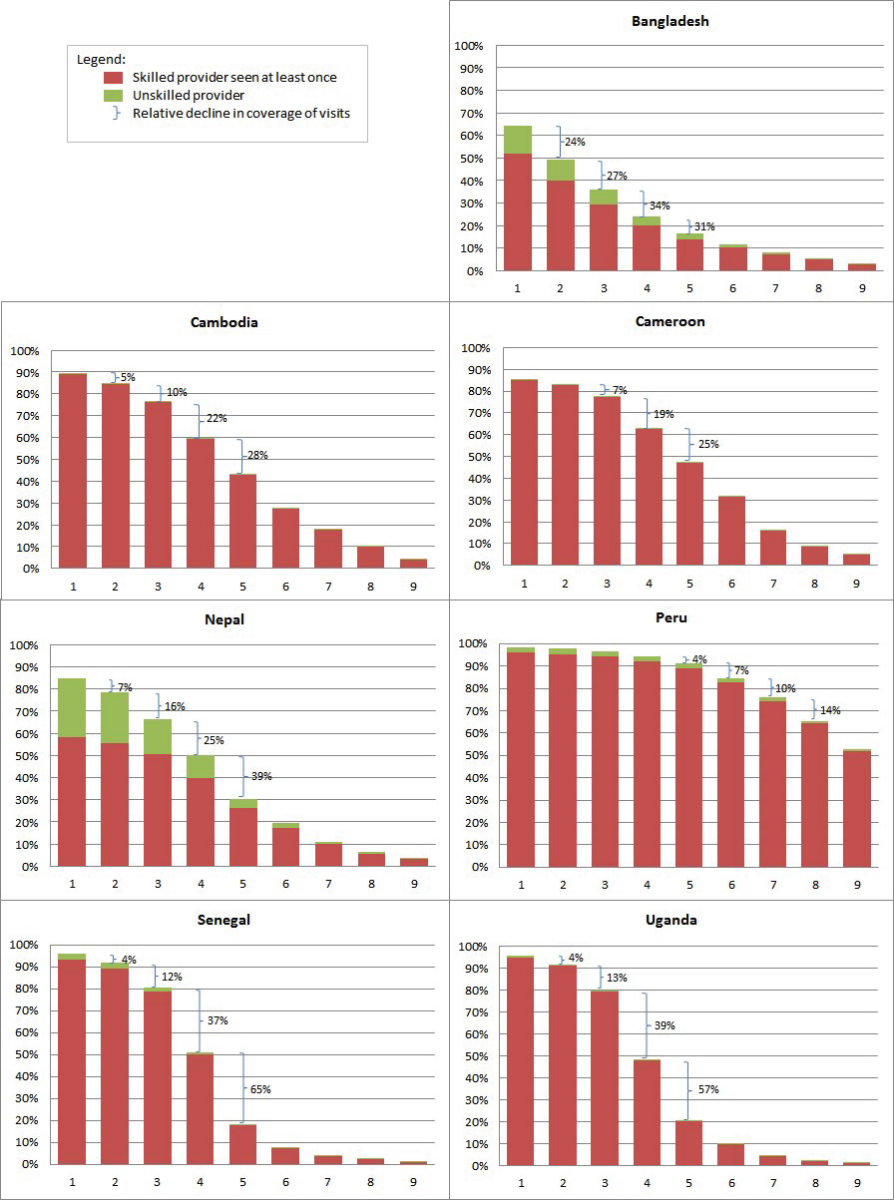
Cumulative percentage of women who had a birth in the five years preceding the DHS surveys by number of ANC visits and type of provider for their most recent live birth, in seven Countdown countries.

We present the cumulative distribution of ANC visits by provider type and the relative decline in proportions of women across the visits in **Figure 3**. In Bangladesh, Cambodia, Cameroon and Peru, the proportions of women who reported receiving ANC appears to gradually decrease as the number of visits increases. In Senegal and Uganda, there seems to be a pronounced drop off between three and four or more visits; in Nepal this noticeable drop off occurs between four and five visits. Similar to the results in **Table 1**, most women reported receiving care from a skilled provider during one or more ANC visits. The relative decline in the proportion of women who reported receiving care from unskilled providers decreased faster than the relative decline of the proportion of women who reported receiving care from skilled providers, as the reported number of visits increased.

**Figure 4:**
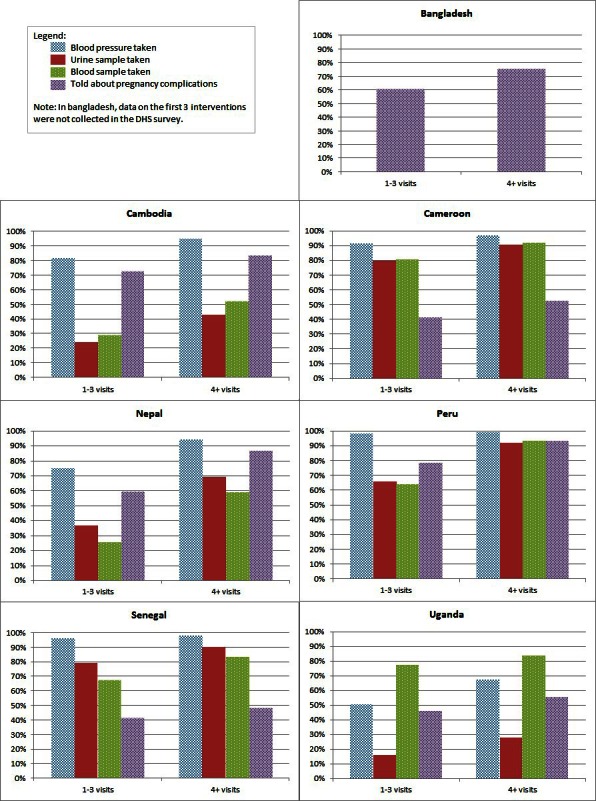
Percentage of women receiving content interventions during any ANC visit among women reporting one to three ANC visits or four or more ANC visits for their last live birth in the five years preceding the DHS survey, in seven Countdown countries.

We show the percentage of women receiving selected content interventions during any ANC visit among women reporting one to three or four or more visits in **Figure 4**. Women who reported four or more visits reported receiving at least one content intervention more than women reporting one to three visits, even though the increase in proportions varied across countries and among interventions; this pattern is also visible after stratifying the percentage of women receiving content by type of provider reported (Table S3 in **Online Supplementary Document(Online Supplementary Document)**) However, a considerable proportion of women who reported the recommended four or more ANC visits did not receive any of the essential interventions at least once. Women who reported receiving ANC services and seeing a skilled provider at least once, seemed to report receiving more content interventions than women who received care from unskilled providers (Table S3 in **Online Supplementary Document(Online Supplementary Document)**). Stratification of women’s reported content by household wealth quintile (Table S4 in **Online Supplementary Document(Online Supplementary Document)**), showed that as women’s wealth status increased, so did their proportions of reporting receipt of content interventions at least once during any visit.

We looked at gestational age at first ANC visit among women who reported receiving ANC visit for their most recent birth in the five years preceding the DHS survey. The results show that women who had their first ANC visit during the first trimester reported a higher number of visits overall (Figure S5 in **Online Supplementary Document(Online Supplementary Document)**). In Cameroon and Uganda, the proportion of women who made their ANC debut during the second trimester is high relative to other countries. Nearly 40% of women in Cameroon and around half of the women in Uganda started ANC during their second trimester. In all seven countries, the proportion of women who report starting ANC in the third trimester is around 5% with the exception of Uganda, where 13% of the women report receiving ANC for the first time in the third trimester; these women generally report three or less visits.

**Figure 5:**
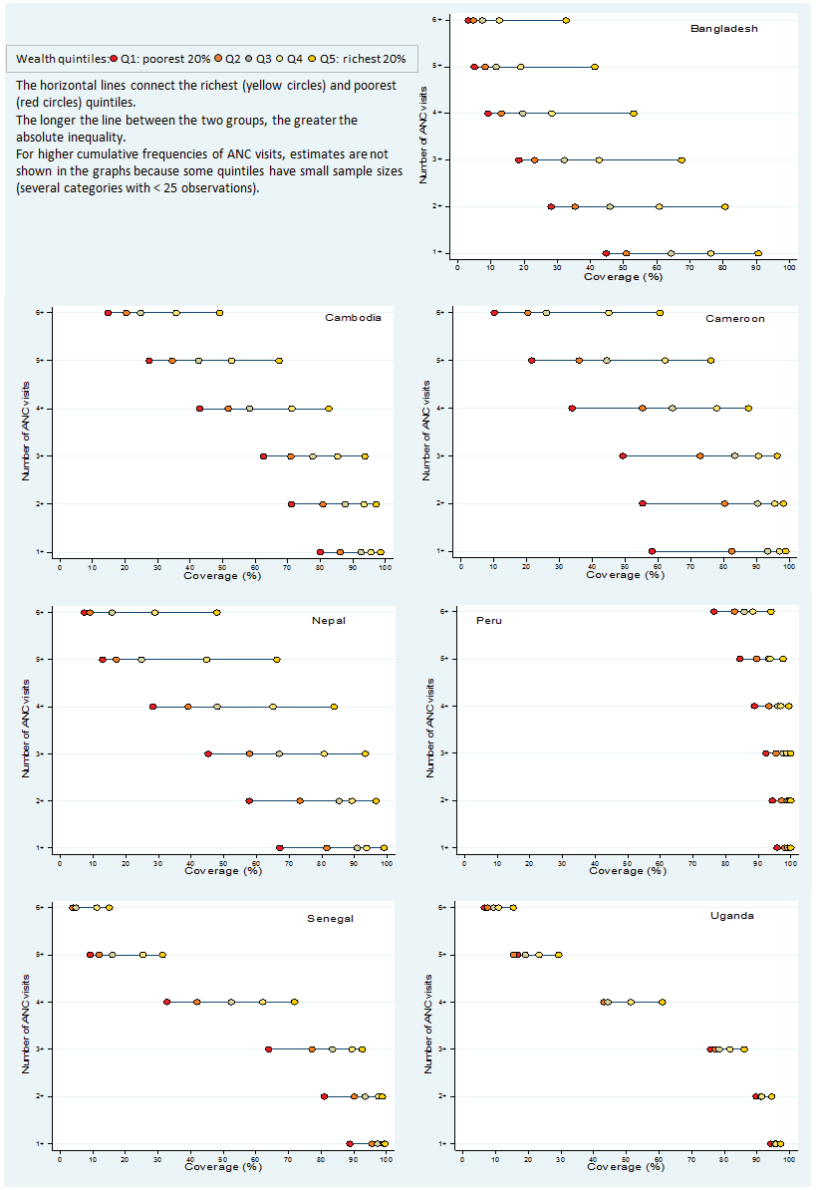
Percentage of women who had a live birth in the five years preceding the DHS surveys by number of ANC visits and household wealth quintiles, in seven Countdown countries.

We present the distribution of women who reported receiving ANC visits by household wealth quintile in **Figure 5**. Wide disparities in the proportions of women reporting utilization exist across the wealth quintiles, except in Peru and Uganda. Top inequality exists where women in the richest wealth quintile are much better off than the rest; bottom inequality exists where women in the poorest wealth quintile are worse off than the rest of the women [35]. Inequality patterns differ by country. In Bangladesh, ANC utilization patterns clearly show top inequality, whereas in Cambodia and Nepal a pattern of top inequality begins to emerge only as the number of visits increases. The greatest disparities among countries are found in the proportions of women’s reported visits by educational level (Figure S6.A in **Online Supplementary Document(Online Supplementary Document)**). Women with the highest level of education report the highest proportions of visits. A pattern of top inequality emerges as the number of visits increases in all countries except Peru, where the inequality by woman’s education is minimal and linear. Inequalities also exist in ANC utilization by place of residence (Figure S6.B in **Online Supplementary Document(Online Supplementary Document)**). In all seven countries, women living in urban areas reported higher proportions of visits compared to their counterparts residing in rural areas. As a result of the drop off in utilization in Senegal and Uganda (**Figure 2**), the proportions of women who reported four or more visits show a considerable decline across all the wealth quintiles, educational levels and by urban–rural residence, in addition to a noticeable widening of the gap across categories of the three stratifiers as the number of visits reaches four or more ANC visits.

### Model–based results

In the multivariable analysis, we sought to identify determinants of ANC initiation in Model A (zero visits vs one or more visits), and of frequency of visits in Model B (one to three visits vs four or more visits). Within each country, determinants predicting initiation of care and frequency of visits differed somewhat, except in Bangladesh where similar determinants predicted the outcome measures in both models at relatively similar odds ratios (OR). We present the results of Model B for the seven countries in **Table 2**; the results of model A and B for each country are found in in Tables S7A–G in **Online Supplementary Document(Online Supplementary Document)**. When we entered the external environment factors, initially, place of residence had a significant effect on ANC in both models in all countries, except Uganda; however, as the subsequent levels were added to the models place of residence became insignificant. The exceptions are Bangladesh and Senegal where women residing in rural areas were less likely to report at least one visit, in Model A (OR: 0.67 and 0.62, respectively) and less likely to report four or more visits in Model B (OR: 0.52 and 0.74, respectively), compared to women residing in urban areas. Generally, among women’s predisposing characteristics, educational level was the strongest predictor of the outcome measures. In Peru, Senegal and Uganda, educational level was significantly positively associated with initiation of care (in Model A) only. In Bangladesh, Cambodia, Cameroon and Nepal, having any level of education significantly increased the odds of initiating ANC and having a higher frequency of visits, compared to having no level of education. Woman’s age at last birth became less significant as the determinants from other levels were added into the Models. The only age group which recorded a significant effect on the outcome measures was the 20–34 years age group, where women in this age group in Cambodia, Nepal and Uganda were more likely to report having at least one ANC visit compared to women who were less than 20 years old (odds ratios ORs: 1.58, 1.54 & 1.62, respectively). While in Cambodia, Cameroon and Peru, women aged 20–34 were more likely to have four or more visits compared to women who were less than 20 years old (ORs: 1.43, 1.57 & 2.48, respectively). The effect of the other ‘predisposing’ characteristics (woman’s occupational status, religion and marital status) were generally not significantly related to women’s reported frequency of visits. Within the enabling resources, current partners’ education affected the outcome measures positively in several countries. In Bangladesh, having a secondary or higher education and in Cambodia and Nepal having a secondary education increased the odds of initiating care (Model A) and the odds of having four or more visits compared to women whose current husbands have no education. Household wealth was the strongest predictor of the outcome measures in this level. Household wealth was positively associated with reporting four or more visits in all countries. The richest quintiles presented odds ratios of 2.4 to 6.1 and 1.7 to 3.1 compared to the poorest in Models A and B, respectively; with Senegal having the highest effect–odds ratio of 7.7 in Model A and Bangladesh having the highest effect–an odds ratio of 4.1 in Model B. Decision regarding the woman’s health care was only significant in Cameroon (Model A), Nepal (both models) and Peru (Model B) and had a negative effect on the outcome measure. In the fourth and final hierarchical level, the “need” factors, women whose gestational age at initial visit was in the second or third trimester were significantly less likely to have four or more visits compared to women who had their initial visit in the first trimester. Birth rank had a significant negative association with initiation of ANC in Cambodia and Nepal; in Bangladesh, Peru and Senegal the negative effect of birth rank is significant at the 5^th^ birth or higher. Birth rank is also negatively associated with the frequency of ANC visits among women’s 5^th^ birth or more in Bangladesh, Cambodia, Nepal, Peru and Senegal. Preceding birth interval was a strong predictor of the outcome measure in both models in Bangladesh, Cambodia, Cameroon and Nepal showing a negative association.

**Table 2:** Multivariable analysis results for Model B (1–3 ANC visits vs 4 or more ANC visits) for seven Countdown countries after hierarchical enter of the determinants into the analysis

Characteristics		Bangladesh			Cambodia			Cameroon			Nepal			Peru			Senegal			Uganda		
**OR**	**95% CI**	***P*–value**	**OR**	**95% CI**	***P*–value**	**OR**	**95% CI**	***P*–value**	**OR**	**95% CI**	***P*–value**	**OR**	**95% CI**	***P*–value**	**OR**	**95% CI**	***P*–value**	**OR**	**95% CI**	***P*–value**
External Environment:*																						
Place of residence	Urban	*Ref*			*Ref*			*Ref*			*Ref*			*Ref*			*Ref*			Exc†	†Exc	†Exc
	Rural	0.52	0.42–0.65	0.000	0.75	0.55–1.02	0.068	0.92	0.71–1.19	0.529	0.88	0.64–1.22	0.454	1.05	0.70–1.58	0.814	0.74	0.61–0.88	0.001	†Exc	†Exc	†Exc
**Predisposing characteristics:**																						
Woman's age at last birth	<20	Exc†	Exc†	Exc†	*Ref*			*Ref*			*Ref*			*Ref*			Exc†	Exc†	Exc†	Exc†	Exc†	Exc†
	20–34	Exc†	Exc†	Exc†	1.43	1.07–1.91	0.017	1.57	1.24–1.97	0.000	1.17	0.88–1.56	0.278	2.48	1.46–4.20	0.001	Exc†	Exc†	Exc†	Exc†	Exc†	Exc†
	35–49	Exc†	Exc†	Exc†	1.48	0.98–2.24	0.064	1.46	1.06–2.01	0.021	1.21	0.70–2.10	0.490	1.67	0.82–3.37	0.155	Exc†	Exc†	Exc†	Exc†	Exc†	Exc†
Woman's educational level	No education	*Ref*			*Ref*			*Ref*			*Ref*			*Ref*			*Ref*			*Ref*		
	Primary	1.58	1.19–2.10	0.002	1.27	1.00–1.61	0.046	1.15	0.88–1.50	0.312	1.75	1.32–2.32	0.000	0.46	0.21–1.03	0.058	1.00	0.83–1.21	0.971	1.05	0.82–1.34	0.721
	Secondary	2.19	1.67–2.87	0.000	1.62	1.21–2.16	0.001	1.56	1.13–2.16	0.007	2.28	1.72–3.01	0.000	0.74	0.31–1.79	0.508	0.84	0.62–1.14	0.265	1.04	0.77–1.40	0.794
	Higher	4.23	2.91–6.13	0.000	2.94	0.84–10.25	0.090	3.89	1.85–8.16	0.000	6.12	2.57–14.58	0.000	0.95	0.32–2.78	0.923	1.94	0.58–6.52	0.281	1.46	0.92–2.33	0.106
Woman's employment status	Did not work in the past 12 months	Exc†	Exc†	Exc†	*Ref*			†Exc	†Exc	†Exc	†Exc	†Exc	†Exc	†Exc	†Exc	†Exc	†Exc	†Exc	†Exc	†Exc	†Exc	†Exc
	Worked in the past 12 months	†Exc	†Exc	Exc†	0.88	0.70–1.11	0.286	†Exc	†Exc	†Exc	†Exc	†Exc	†Exc	†Exc	†Exc	†Exc	†Exc	†Exc	†Exc	†Exc	†Exc	†Exc
Religion	Dominant religion	*Ref*			*Ref*			†Exc	†Exc	†Exc	†Exc	†Exc	†Exc	†Exc	†Exc	†Exc	†Exc	†Exc	†Exc	†Exc	†Exc	†Exc
	Other religions	1.25	0.97–1.61	0.090	0.51	0.30–0.86	0.012	†Exc	†Exc	†Exc	†Exc	†Exc	†Exc	†Exc	†Exc	†Exc	†Exc	†Exc	†Exc	†Exc	†Exc	†Exc
Marital status	Currently in union	†Exc	†Exc	Exc†	†Exc	†Exc	†Exc	*Ref*			†Exc	†Exc	†Exc	*Ref*			†Exc	†Exc	†Exc	†Exc	†Exc	†Exc
	formerly/Never in union	†Exc	†Exc	Exc†	†Exc	†Exc	†Exc	0.84	0.60–1.18	0.318	†Exc	†Exc	†Exc	1.29	0.49–3.36	0.607	†Exc	†Exc	†Exc	†Exc	†Exc	†Exc
**Enabling factors:**																						
Current partner's educational level	No education	*Ref*			*Ref*			*Ref*			*Ref*			*Ref*			*Ref*			†Exc	†Exc	†Exc
	Primary	1.09	0.87–1.38	0.445	1.16	0.88–1.53	0.283	1.06	0.82–1.36	0.677	1.44	1.05–1.98	0.024	3.20	1.39–7.37	0.006	1.11	0.89–1.38	0.340	†Exc	†Exc	†Exc
	Secondary	1.37	1.09–1.73	0.008	1.59	1.16–2.18	0.004	1.18	0.87–1.59	0.288	1.56	1.12–2.18	0.009	2.48	1.04–5.92	0.041	1.23	0.91–1.67	0.176	†Exc	†Exc	†Exc
	Higher	1.95	1.47–2.59	0.000	2.13	1.17–3.87	0.014	1.20	0.73–1.96	0.466	1.28	0.81–2.03	0.292	2.10	0.67–6.62	0.205	1.85	1.16–2.94	0.010	†Exc	†Exc	†Exc
Current partner's most recent employment status	No recent occupation	†Exc	Exc†	Exc†	Exc†	Exc†	Exc†	Exc†	Exc†	Exc†	**N/A‡**	**N/A‡**	**N/A‡**	**N/A‡**	**N/A‡**	**N/A‡**				**N/A‡**	**N/A‡**	**N/A‡**
	Recently had an occupation	Exc†	Exc†	Exc†	Exc†	Exc†	Exc†	Exc†	Exc†	Exc†	**N/A‡**	**N/A‡**	**N/A‡**	**N/A‡**	**N/A‡**	**N/A‡**				**N/A‡**	**N/A‡**	**N/A‡**
Household wealth quintile	Poorest	*Ref*						*Ref*			*Ref*			*Ref*			*Ref*			*Ref*		
	Poorer	1.16	0.87–1.54	0.325	1.23	0.97–1.55	0.089	1.17	0.93–1.46	0.176	1.04	0.77–1.41	0.795	1.36	0.92–2.02	0.122	1.17	0.99–1.38	0.061	1.16	0.90–1.49	0.244
	Middle	1.55	1.18–2.02	0.002	1.07	0.81–1.40	0.633	1.19	0.89–1.59	0.241	1.05	0.72–1.54	0.800	1.41	0.75–2.66	0.282	1.42	1.16–1.73	0.001	1.23	0.94–1.61	0.125
	Richer	2.15	1.63–2.83	0.000	1.59	1.19–2.12	0.002	1.60	1.13–2.26	0.008	1.55	1.04–2.33	0.033	1.29	0.58–2.85	0.529	1.57	1.20–2.06	0.001	1.66	1.21–2.29	0.002
	Richest	4.08	2.97–5.60	0.000	1.71	1.20–2.44	0.003	1.84	1.24–2.73	0.002	2.36	1.44–3.85	0.001	3.07	0.82–11.49	0.095	1.95	1.42–2.68	0.000	1.98	1.42–2.77	0.000
Decision regarding woman's health care	Woman alone	Exc†	Exc†	Exc†	Exc†	Exc†	Exc†	Exc†	Exc†	Exc†	*Ref*			*Ref*			Exc†	Exc†	Exc†	Exc†	Exc†	Exc†
	Woman & partner	Exc†	Exc†	Exc†	Exc†	Exc†	Exc†	Exc†	Exc†	Exc†	0.93	0.72–1.19	0.554	0.81	0.53–1.25	0.347	Exc†	Exc†	Exc†	Exc†	Exc†	Exc†
	Partner alone	Exc†	Exc†	Exc†	Exc†	Exc†	Exc†	Exc†	Exc†	Exc†	0.72	0.53–0.99	0.041	0.65	0.42–0.99	0.047	Exc†	Exc†	Exc†	Exc†	Exc†	Exc†
	Someone else/other	Exc†	Exc†	Exc†	Exc†	Exc†	Exc†	Exc†	Exc†	Exc†	0.69	0.52–0.91	0.010	0.33	0.06–1.82	0.200	Exc†	Exc†	Exc†	Exc†	Exc†	Exc†
**Need factors:**																						
Gestational age at initial visit	First trimester	**N/A‡**	**N/A‡**	**N/A‡**	*Ref*			*Ref*			*Ref*			*Ref*			*Ref*					
	Second trimester	**N/A‡**	**N/A‡**	**N/A‡**	0.23	0.20–0.27	0.000	0.24	0.20–0.28	0.000	0.36	0.29–0.46	0.000	0.18	0.12–027	0.000	0.29	0.25–0.35	0.000	0.26	0.21–0.32	0.000
	Third trimester	**N/A‡**	**N/A‡**	**N/A‡**	0.02	0.01–0.04	0.000	0.03	0.02–0.04	0.000	0.02	0.01–0.06	0.000	0.01	0.01–0.02	0.000	0.08	0.04–0.15	0.000	0.05	0.03–0.08	0.000
Birth rank	1^st^ – 2^nd^ births	*Ref*			*Ref*			Exc†	Exc†	Exc†	*Ref*			*Ref*			*Ref*			Exc†	Exc†	Exc†
	3^rd^ – 4^th^ births	0.87	0.72–1.04	0.128	0.85	0.69–1.05	0.124	Exc†	Exc†	Exc†	0.85	0.65–1.11	0.223	0.72	0.45–1.14	0.156	0.87	0.73–1.04	0.121	Exc†	Exc†	Exc†
	5^th^ births or more	0.43	0.29–0.64	0.000	0.55	0.40–0.76	0.000	Exc†	Exc†	Exc†	0.58	0.37–0.91	0.018	0.53	0.30–0.93	0.026	0.77	0.65–0.91	0.003	Exc†	Exc†	Exc†
Preceding birth interval	First birth	*Ref*			*Ref*			*Ref*			*Ref*			Exc†	Exc†	Exc†	Exc†	Exc†	Exc†	Exc†	Exc†	Exc†
	Less than 2 years	0.71	0.51–0.97	0.033	0.75	0.55–1.03	0.076	0.64	0.47–0.86	0.004	0.60	0.42–0.86	0.005	Exc†	Exc†	Exc†	Exc†	Exc†	Exc†	Exc†	Exc†	Exc†
	2–3 years	0.62	0.48–0.81	0.000	0.82	0.63–1.08	0.156	0.72	0.55–0.95	0.022	0.69	0.51–0.95	0.021	Exc†	Exc†	Exc†	Exc†	Exc†	Exc†	Exc†	Exc†	Exc†
	More than 3 years	0.80	0.68–0.95	0.010	0.71	0.56–0.91	0.005	0.76	0.59–0.98	0.032	0.84	0.65–1.10	0.215	Exc†	Exc†	Exc†	Exc†	Exc†	Exc†	Exc†	Exc†	Exc†
Pregnancy wanted at the time	Then	Exc†	Exc†	Exc†	Exc†	Exc†	Exc†	Exc†	Exc†	Exc†	Exc†	Exc†	Exc†	Exc†	Exc†	Exc†	Exc†	Exc†	Exc†	Exc†	Exc†	Exc†
	Later	Exc†	Exc†	Exc†	Exc†	Exc†	Exc†	Exc†	Exc†	Exc†	Exc†	Exc†	Exc†	Exc†	Exc†	Exc†	Exc†	Exc†	Exc†	Exc†	Exc†	Exc†
	No more	Exc†	Exc†	Exc†	Exc†	Exc†	Exc†	Exc†	Exc†	Exc†	Exc†	Exc†	Exc†	Exc†	Exc†	Exc†	Exc†	Exc†	Exc†	Exc†	Exc†	Exc†

ANC – antenatal care, OR – odds ratio, 95% CI – 95% confidence interval, Ref – reference category, Exc – excluded, N/A – not available

*Results related to the determinant “region of the country” can be found in Tables 7A–G in **Online Supplementary Document(Online Supplementary Document).**

†Excluded by backward elimination during hierarchical entry at the level or due to insignificance at the bivariate level.

‡Country data was not available for determinant.

### Descriptive review of ANC–related national policies and programs

Each of these seven countries has its own set of national policies, strategies and guidelines around health–related issues and ANC, specifically. **Tables 3** and **4** summarize the information obtained through the questionnaire. Countries vary widely in terms of their ANC–related policies, programs, standards, and guidelines. Here we use selected examples to explore how these variations may have affected the ANC utilization patterns presented in the descriptive and multivariable analysis above. We describe this link with caution, because our data sets are not sufficiently complete or quantitative to determine directional causality.

**Table 3:** Summary of policies and programs related to ANC, in seven Countdown countries

	Selection of national policies and their reference to ANC	Policies & efforts to tackle inequities	ANC–related campaigns or communication efforts
Bangladesh	*National Maternal Health Strategy*–2001: • Specifies the supply of equipment for ANC, the delivery of care and a monitoring checklist. • Outlines interaction with pregnant women and their families to ensure ANC uptake and popularization of service delivery mechanisms as well as the use of ANC for birth preparedness. *Health, Population and Nutrition Sector Development Plan (HPNSDP), 2011–2016:* • ANC mentioned as a key service in emergency obstetric care needs and management. • Specifically mentions distribution of folic acid/and iron supplementation. *National Plan of Action for Adolescent Sexual and Reproductive Health*, 2013: • Specifically published to tackle the major concern of teenage pregnancies which make up around 30% of adolescents aged 15–19 y old.	The *HPNSDP*–2011–2016 prioritizes the improvement of ANC by: • Emphasizing maternal, newborn, child and adolescent health interventions/services in urban slums, hard to reach and low performing areas. • Prioritizing areas of high maternal mortalities and geographically & socially disadvantaged population.	Available through various types: • Television and radio programs. • Mass communication during the Safe Motherhood days when ANC is promoted along with other services. • Posters and pamphlets available at health facilities.
Cambodia	*Health Sector Strategic Plan for 2008–2015:* • Plans to scale up access to and coverage of health services, especially comprehensive reproductive, maternal, newborn and child health services. *Safe Motherhood Clinical Management Protocols for Referral Hospital*–June 1013 & *Safe Motherhood Clinical Management Protocols for Health Centre*–July 2010: • Provide technical updates regarding frequency of ANC visits (from 2+ to 4+), timing (as early as knowing the missing period), and additional services (screening).	The *Health Sector Strategic Plan* – 2008–2015 has pro–poor policies including: • Pro–poor health financing systems • Exemptions for the poor • Expansion of the health equity funds • Health insurance	To promote early ANC visits an ANC campaign took place in 2009 using both mass media and interpersonal communication in selected geographical areas.
Cameroon	Prenatal care centres (*Soins Prenataux Recentres*)–2006 • Includes change from the focus on the number of visits to the quality of the visit. *Prevention of Mother to Child Transmission of HIV*–November 2008	A project initiated by the World Bank in 14 districts to test performance–based financing addresses some aspects of inequity.	No specific efforts noted
Nepal	*National Health Policy* – 1991: • Adopted the safe motherhood approach with *the Safe Motherhood Program* being a priority. *Safe Motherhood Policy*–1996: • Focuses on improving maternal health in a holistic way *National Policy on Skilled Birth Attendants NPSBA)*–2006 *National Medical Standard for Reproductive Health*–2009*:* • An updated version of the *National Maternity Care Guidelines (NMCG)* which was released in 2006. • A standard reference document for essential clinical materials and tools in support of patient care using the latest evidence in maternal and neonatal care. • Uses the concepts of focused antenatal care. • Emphasis is on every pregnant woman being at risk, birth preparedness and complication readiness, providing quality rather than quantity of antenatal care.	No specific efforts noted.	• The Government implemented a communication strategy. • ANC–related messages are broadcast through radio.
Peru	*Comprehensive Health Insurance* (*The Seguro Integral de Salud (SIS)) – 2001*: • Aims to protect the health of Peruvians who do not have health insurance, prioritizing those vulnerable populations who are in poverty or extreme poverty. *Budgeting for results (Presupuesto por Resultados)* – 2008: • It proposes action based on critical problem solving and includes Strategic Programs such as the one for Mother and Newborn (which was established for women in extreme poverty & no health insurance). *Technical document: National Strategic Plan for Reduction of Maternal and Neonatal Mortality (RN No. 207–2009)* *Technical Guides: Intervention Model to improve Access, Quality and Use of facilities that provide obstetric and neonatal functions (RM No. 223–2009/MINSA)* • provides strategies to improve availability, accessibility and use of facilities. *Technical Standard for the comprehensive care of maternal health (RM No. 827–2013/MINSA)* • Establishes the technical requirements and administrative procedures, based on scientific evidence, that allow to deliver quality care in preparation for pregnancy, refocused prenatal care, institutional and skilled delivery care and postpartum care.	• The *Seguro Integral de Salud (SIS)*–2001 • The *Mother and Newborn Strategic Program* • The *Technical Standard for Vertical Delivery with Attention to Intercultural Adaption*: intended to improve access for Andean and Amazonian women of childbearing age. • The establishment of Maternal Waiting homes to increase access to delivery care in health facilities.	Different media used to communicate importance of ANC
Senegal	*National Program for the Prevention of Maternal Mortality (Programme Nacional de Prevention de la Mortalite Maternelle)*–1990 *Politique de Santé et d’Action Sociale* (Health Policy and Social Action)–1995 • Placed reproductive health as one of the cornerstones. *Population Policy Statement (Déclaration de Politique de Population)* • Established in 1998 & updated in 2002 to be consistent with the ICPD. *National Program of Reproductive Health (Programme Nacional de santé de la Reproduction)*–2002 *A multi–sectoral roadmap* • Developed to accelerate the reduction of maternal and neonatal mortality in order to achieve MDGs 4 & 5.	The national strategy for all women of reproductive age has elements for making services available to all–geographically, financially, socio–culturally, and to all religious groups through: • Increasing points of service delivery. • Provision of minimum package of reproductive health services at health facilities. • Adjusting the fees/costs according to people’s abilities to pay.	• Government conducted nationwide scale up campaign with radio and television spots on malaria prevention with SP and use of ITNs. • NGOs support this campaign by broadcast general messages on antenatal care through local–community radio.
Uganda	*The National Policy Guidelines and Service Standards for Sexual and Reproductive Health and Rights–*2012 (3^rd^ update) • Sets rules and regulations governing reproductive health services including antenatal services • Outlines tasks that guide service provision and describe aspects of ANC services • Emphasizes integration of services such as access to services for sexually transmitted infections and HIV/AIDS services at the ANC clinic *Integrated National Guidelines on Antiretroviral Therapy, Prevention of Mother to Child Transmission and on Infant & young Child feeding*–2011 • Facilitates integration of services and to promote a family–centered approach for HIV and AIDS care and treatment. • ANC is recognized as a platform for this care and treatment.	A voucher scheme for pregnant women is being piloted in a few areas.	• Radio messaging on particular aspects of ANC, eg, malaria prevention and prevention of mother to child transmission of HIV • Some projects in limited geographic areas have used phone text messages to ANC clients

ANC – antenatal care, SP – sulfadoxine–pyrimethamine, ITN – Insecticide–treated bednet, NGO – non–governmental organization, ICPD – International Conference on Population and Development, MDG – Millennium Development Goals, HIV – human immunodeficiency virus, AIDS – Acquired Immune Deficiency Syndrome.

**Table 4:** Summary of national standards and guidelines for ANC, in seven Countdown countries

	Recommended number of ANC visits	Presence of guidelines for visit content	Where ANC services are provided	Who provides the ANC services	Presence of user fees	Incentives for women’s utilization
**Bangladesh**	Follows the WHO recommendation: • 1^st^ visit: before 16 weeks. • 2^nd^ visit: 24–28 weeks. • 3^rd^ visit: 30–32 weeks. • 4^th^ visit: 36–38 weeks.	Present	Provided at both private sector and public sector (primary, secondary and tertiary facilities) and through NGOs. Home–based ANC may be provided in rural areas.	In urban areas and the private sector, doctors usually provide ANC. In rural areas there is a wider array of skilled and unskilled providers who offer ANC services.	No public sector fees. Private facilities charge fees for service.	In some Upazila Health Centers (public facilities) patients receive transportation cost.
**Cambodia**	Follows the WHO recommendation: • 1^st^ visit: before 16 weeks (or as soon as possible after a missed menstrual period). • 2^nd^ visit: 24–28 weeks. • 3^rd^ visit: 30–32 weeks. • 4^th^ visit: 36–38 weeks.	Present	Provided at health centers (primary facilities) or hospitals (tertiary level).	Types of providers & services are the same in urban and rural public facilities. Services in private facilities depend on ability to pay. ANC services are generally provided by midwives.	Public facilities have user fee schemes. Private sector facilities have a fee–for–service.	Some schemes offer indirect incentives through: • Health equity fund • Voucher scheme linking ANC services to other MCH services.
**Cameroon**	Recommends four visits: • 1^st^ visit at 1–16 weeks amenorrhea. • 2^nd^ visit at 28 weeks. • 3^rd^ visit at 32 weeks. • 4^th^ visit at 36 weeks.	None provided	Present at all health facilities.	Providers do not vary according to public/private sector or to rural/urban areas. ANC services are provided by various skilled & unskilled providers and at various workstations in one facility.	Both the public and private sector charge fees at different rates.	No incentives available
**Nepal**	Recommends four visits: • 1^st^ visit at 4 months • 2^nd^ visit at 6 months • 3^rd^ visit at 8 months • 4^th^ visit at 9 months	Present	In rural areas, ANC is provided at sub–health posts, health posts and district hospitals. In urban areas, ANC is provided at private clinics and maternity hospitals.	All service providers should be skilled birth attendants (these include nurses and doctors). If these skilled providers are not available at Sub–health posts and out–reach clinics, then MCH Workers can provide ANC services.	No public sector fees. Private sector charges vary.	Incentives provided to women who complete 4 ANC visits and have an institutional delivery.
**Peru**	Recommends a minimum of 6 visits: • 1^st^ visit: at less than 14 weeks • 2^nd^ visit: 14–21 weeks • 3^rd^ visit: 22–24 weeks • 4^th^ visit: 25–32 weeks • 5^th^ visit: 33–36 weeks • 6^th^ visit: 37–40 weeks	Present	Most ANC services are provided through the network of 8000 public facilities. Home visits are made when women miss their scheduled visit.	Service providers are mainly skilled. Unskilled providers are usually involved in the health team particularly at the first level of the health system.	Fees depend on different funding sources.	Specific program created in 2005, provides program grants for direct transfers to benefit the poorest families, rural and urban.
**Senegal**	Recommends at least 4 visits: • 1^st^ visit at 3 months • 2^nd^ visit at 6 months • 3^rd^ visit at 8 months • 4^th^ visit at 9 months	Present	Provided through health huts, health posts, maternity centers or hospitals and private clinics.	Standards and protocols stipulate that only skilled providers can provide ANC services at both public and private facilities	Both public and private sector facilities charge fees but at different rates.	Insecticide–treated bednets are provided to pregnant women.
**Uganda**	Recommends four focused ANC visits: • 1^st^ visit: 0– 16 weeks (after two missed periods). • 2^nd^ visit: 16–28 weeks. • 3^rd^ visit: 28–36 weeks. • 4^th^ visit: after 36 weeks. • 4^th^ visit: after 36 weeks.	Present	Provided at hospitals, health centers, and sometimes at outreach clinics.	Skilled providers provide ANC services in all facilities. Unskilled providers such as community health workers & village health team members can provide information. Nursing assistants & nurse aids are being phased out.	No public sector fees. In Private not–for–profit facilities fees are subsidized. Private for profit sector generally does not subsidize ANC except for immunization.	Mama Kit of essential supplies to use during delivery (gloves, protective sheets, baby receiving sheet, soap) are provided to pregnant women.

ANC – antenatal care, WHO – World Health Organization, NGO – non–governmental organization, MCH – maternal and child health.

Bangladesh and Peru, at the two extremes of ANC 1+ and ANC 4+ coverages among these seven countries, have comprehensive guidelines and policies related to ANC. Although, ANC utilization is relatively low in Bangladesh, trends in coverage of ANC 1+ and ANC 4+ have been increasing steadily since the early 1990s [13]. Unlike Bangladesh and the other five countries, the Peruvian government goes beyond the WHO guidelines of four ANC visits and recommends a minimum of six scheduled visits.

Both Senegal and Uganda show a distinct drop–off in ANC utilization between the third and fourth visits. In Senegal, several reproductive health–related policies were either updated or developed between 2002 and 2005, and one of the changes included moving from a standard of three to four ANC visits. In Uganda, the government has adopted a four–visit, focused ANC approach, and recently introduced guidelines addressing HIV/AIDS and prevention of mother–to–child transmission that refers to ANC as a platform for care and treatment. However, the reported number of visits by gestational age during the first ANC visit (Table S5 in **Online Supplementary Document(Online Supplementary Document)**) showed that 66% and 13% of Ugandan women report initiating care during their second and third trimester, respectively, which inevitably means there is less time to complete the recommended number of visits prior to childbirth. The results of Uganda’s multivariable analysis also show that as the gestational age at first ANC visit increases, women are significantly less likely to report receiving four or more ANC visits compared to three or fewer.

In Nepal, the proportion of women receiving care only from an unskilled provider was the highest among the seven countries, followed by Bangladesh. Our contextual information showed that both these countries had clear guidelines permitting unskilled providers to offer certain ANC services. The *National Medical Standards for Reproductive Health* guideline, adopted by the Nepali government in 2009, states that in the absence of a skilled birth attendant in the facilities serving rural areas, a maternal and child health worker or a health assistant (categorized as unskilled providers in our study) can provide ANC services [36]. With 90% of our sample of Nepali women residing in rural areas, high proportions of women may have only had access to unskilled providers offering ANC services. Furthermore, some reports from Nepal refer to unskilled providers such as health assistants, auxiliary health workers, maternal and child health workers, and village health workers as trained professionals [37]. In Bangladesh, similar to Nepal, the majority of women reside in rural areas (around 75% of our sample), which are served by a complex network of public health facilities offering ANC services by skilled and unskilled providers. At community level, providers now considered unskilled for ANC, historically provided services at primary facilities and household level through both government and non–government agencies [38].

## DISCUSSION

The globally–measured ANC indicators, ANC 1+ and ANC 4+, need to be accompanied by more detailed analysis of ANC utilization patterns in each country in order to unpack the underlying factors and inequalities that play a role in women’s uptake of ANC services.

We intentionally selected countries for analysis from different world regions and with varying levels of ANC 1+, ANC 4+ and skilled birth attendance coverage. Skilled birth attendance is lowest, at 32% and 36%, in Bangladesh and Nepal, respectively [13]; in addition to having the lowest proportions of one or more and four or more ANC visits, our results also showed that these two Asian countries reported the highest prevalence of ANC provision by an unskilled provider. However, local definitions of what constitutes a skilled provider seem to vary in these two countries as described in the descriptive review of national policies and programs.

Nevertheless, the majority of women in the seven Countdown countries reported receiving care from a skilled provider at least once. The reported content interventions, on the other hand, require more attention. Even in Peru, where over 90% of women reported receiving four or more visits, evidence–based content was highest relative to other countries yet not universal. Regardless of countries’ diverse settings, women who reported four or more ANC visits, who received care from a skilled provider at least once and were better off in terms of household wealth, reported receiving a higher proportion of each of the four content interventions. Similar results were also presented by Hodgins et al (2014), who looked at DHS data on content interventions of ANC visits from countries [31]. In their analysis, the proportion of content interventions (out of eight) among women who reported four or more visits ranged from 32% to 85% in the 41 countries and the overall average was 60% [31]. These relationships need to be explored further at the country level to understand whether content interventions are not being provided during ANC visits or are being postponed to later during the pregnancy, resulting in missed opportunities for women who report a low number of visits. Or, on the contrary, the perceived usefulness and quality of the interventions offered at health care facilities may play a role in women’s decisions about whether to return for subsequent visits. We did not consider the health care facilities providing the ANC services in our quantitative analysis, yet this may play an important role in women’s ANC utilization patterns especially if the quality of care is perceived as poor. Powell–Jackson and colleagues looked at the quality of ANC services in the private commercial sector, private not–for–profit sector, public sector and home from DHS data in 46 low– and middle–income countries [39]. The content of care score was worst in home–based care, where women received the least number of ANC services, followed by both the public and private commercial sectors with similar scores and the private not–for–profit had the highest ANC content score. The researchers conclude that the private commercial and public sectors are both very diverse and show lots of variation in quality of care, possibly depending on the economic status of women seeking ANC care [39].

In order to ensure high coverage of accessible and equitable ANC services, programs and policies need to focus on women with low levels of education, living in poor and rural households. Our findings indicate that household wealth is an important determinant of ANC initiation in all seven countries, and of the overall frequency of visits in all countries except Peru. This is consistent with a systematic review of the relevant literature [27]. Our multivariable analysis results showed that women who come from poorer households are less likely than richer women to initiate care, and among those who do seek ANC, less likely to have four or more visits. These multivariable results complement the results of the equity analysis, and highlight the need to address financial barriers to accessing ANC services. ANC services are offered by the public sector free of charge in Bangladesh, Nepal and Uganda, and yet utilization is relatively low. Unexpected fees for prescribed medications or tests, and indirect costs related to transportation to the facility, have been associated with women’s choices of health care services, and need to be considered [28]. The exceptional case of Peru may reflect its unique combination of political will, economic growth, broad societal participation, pro–poor strategies and increased spending in health and related sectors in the last two decades, which led to reduction in socioeconomic inequalities in health and significant progress in coverage of RMNCH interventions especially among the most deprived groups and areas of the country [40].

Education allows women to be more autonomous, more knowledgeable about health care services, and therefore to exert greater control over health–related decisions. We would therefore expect women’s education to have a positive influence on the initiation and frequency of ANC visits [19,20,27], and this is supported by our results. The wide disparities in ANC utilization by levels of women’s education may also be due to the uneven distribution of women within each category; women who have attained higher levels of education are fewer in number and generally better off than those who report having low or no education. The results of the multivariable analysis showed that education was a significant determinant for initiating ANC, and to a lesser extent, for reporting four or more ANC visits. Similar to the results of Guliani and colleagues [19], who looked at the use of ANC services and their frequency across 32 low income countries, the association of women’s education was stronger with the initiation of ANC visits than with the overall number of visits. This may be because women with no education are not included in the second model, which looks at the frequency pattern of utilization [19].

The equity analysis showed that women residing in rural areas have lower proportions of ANC utilization than women residing in urban areas, and our policy data suggested important differences in services and providers in urban and rural areas in most country settings. We were therefore surprised that urban–rural residence emerged as a significant determinant of ANC visits only in Bangladesh and Senegal. A systematic review looking at early use of ANC services and type of residence concluded that the association was not consistent [41], hence further country analysis is required to identify the contextual factors that affect ANC use. A study looking at contextual influences of 13 sub–regions in Nepal on women’s ANC patterns identified important sub–regional variations in ANC use, which need to be taken into consideration at the policy–making level [42]. Our findings reinforce the importance of regional differences in ANC utilization within countries, and suggest that further analysis of this relationship is likely to generate information useful for ANC program planning.

We collected some information on barriers to ANC utilization from national surveys or ethnographic studies through our key informant interviews, but were often unable to obtain full and relevant information. Qualitative studies exploring barriers to antenatal care are available in the literature, and can contribute to the interpretation of our findings. In Bangladesh, despite multiple maternal health policies and an extensive array of public sector ANC facilities, women are not utilizing ANC services as recommended. This has been attributed by some to women’s perception that pregnancy is a normal event that does not need medical care and interventions [43]. In Cambodia, five types of barriers to maternal health care use have been identified as needing to be addressed to increase ANC utilization: financial, physical, cognitive, organizational and psychological/socio–cultural [44]. One study reported that the use of ANC services by pregnant women in Nepal was greatly influenced by mothers–in–law [45]; this is corroborated by our multivariable results showing a negative association between women’s reports that they are not responsible for health care decisions and the frequency of visits. Another study reports that mothers in Uganda viewed ANC services as deficient, and are dissatisfied with the perceived quality of the interventions offered during visits [46]. WHO has identified several barriers to the provision of quality ANC, including perceptions of poor quality of care, distance, cost, stigma, social and traditional influences, perceptions that pregnancy is a healthy state that does not need specific care, and disrespect for and abuse of women in health service settings [47]. Furthermore, a systematic scoping review performed to understand what women seek during pregnancy, found that across diverse settings, having a positive pregnancy experience was what mattered to pregnant women and this was characterized by four themes: preserving physical and sociocultural normality; maintaining a healthy pregnancy for mother and baby; effective transition to progressive labour and birth; and ensuring positive motherhood [48].

Our study has some limitations that need to be kept in mind when interpreting the results. DHS data are collected on ANC–related questions, only, for women’s most recent live birth in the five years preceding the survey. Hence, pregnancies resulting in a miscarriage or a stillbirth are excluded and no data on the ANC utilization patterns of these pregnancies is collected; data which may provide important insights to the patterns and quality of care in these cases specifically. Furthermore, the DHS data we use is from interviews with women and their responses to the frequency of ANC visits, types of providers seen, and content interventions received for their most recent live birth. As a result, there may be potential recall bias, an issue which is receiving increased awareness in mothers’ reports of services received [49]. Women may also be biased in their reports of the type of provider from whom they received care, especially in settings where several types of providers offer ANC care. Other limitations related to the type of provider are the fact that the DHS data cannot tell us the type of provider visited during each of the woman’s ANC visit and that the choice of providers may be restricted to who is available at the health care facility in the community. We report on content received during any ANC visit, and while it does imply a minimum level of quality of care, we are unable to confirm it, because the DHS does not assess whether the content interventions were offered in a proper and timely way. Women are also asked to report on their own and their partner’s employment status at the time of the survey interview, and this may have changed since the time of their most recent pregnancy. The DHS does not include questions that are directly related to the barriers to seeking ANC or accessing ANC facilities. However, we used the question about who makes decisions about the woman’s health care as a measure of one potential barrier to access of services. Our findings do not reflect other potential barriers such as distance to a facility or associated financial costs. Another limitation is that the data we were able to obtain on policies and programs in each country varied greatly, and because most of our key informants volunteered their time, it was not always possible to confirm all details or to seek additional information and clarification within the time frame of this study.

Nevertheless, this analysis has several strengths. We examine ANC utilization from a new perspective, focusing specifically on information missed by global tracking of only two indicators. The study brings together several types of analysis–descriptive, equity, and inferential analysis–to generate new and detailed results of specific characteristics of women and their households that are associated with ANC utilization patterns in seven diverse countries.

## CONCLUSION

The results of this study indicate that reporting the globally measured ANC indicators, ANC 1+ and ANC 4+, is useful to provide an overall idea of the proportions of ANC utilization in countries. However, descriptive and multivariable analyses generated a much better understanding of each country’s unique pattern of ANC utilization, as well as the characteristics of women not currently receiving adequate care. The presence of variations across countries suggests the need for specific in–country assessments, national panels, or advisory groups to look more closely at national data, commission specific studies and perhaps try different models of ANC to find ways to achieve universal ANC coverage.

A number of predominant aspects of ANC utilization patterns emerged across the seven Countdown countries. Our results highlight the need to focus on evidence–based content interventions offered to women during their ANC visits. Further quantitative assessments of the frequency and timeliness of content interventions by different types of providers and in different settings are needed to ensure proper administration of the WHO–recommended interventions. Moreover, qualitative studies looking into the barriers of ANC use and women’s perceptions of ANC services in each country are essential. There is a growing body of literature that focuses on women’s perceptions of pregnancy and quality of ANC services and how important this aspect is on ANC uptake. The current DHS protocol asks about barriers to seeking health care in general; it may provide important insights to include a question specifically about the numerous barriers that may affect women’s initiation of ANC and completion of the four recommended visits.

Inequality in ANC utilization patterns among women of different wealth statuses, educational backgrounds and places of residence need to be considered at the policy–making level across most of the countries we studied. These dimensions of inequality were strong predictors of ANC utilization and higher frequency of visits, except for place of residence. The influence of place of residence on ANC utilization in Bangladesh and Senegal suggests the need to assess the health care services offered in rural areas. And the lack of significance of this factor in the five other countries suggests that there are variations within each place of residence that need to be identified and used to provide effective interventions. While we found that policies and guidelines related to ANC as well as ‘Safe Motherhood’ strategies were incorporated into the national policies, across the seven countries, yet, there is a need to ensure evaluation and proper implementation of these policies and strategies. Peru is an example of successful implementation of political and structural reform, which took place in the 1990s, and led to the enhancement of health systems and infrastructure, reduction in poverty, and the introduction of insurance schemes, one of which is for pregnant mothers, among other groups [50].

With the end of the MDG era, few countries have achieved the MDG5 goal of reducing maternal mortality by three quarters, and most have a long way to go before achieving universal access to reproductive health services [13]. Most maternal deaths are preventable, and the causes of these deaths are known [2,47,51]. By increasing attention and investment to providing quality maternity, antenatal, and post–partum care, life–saving interventions may be administered properly and in a timely manner by skilled health providers to help improve maternal and neonatal health and their survival [2]. In the post–2015 agenda, as the Sustainable Development Goals and their measurable indicators are being set, it is essential to include targets for ending preventable maternal deaths and to ensure that the momentum focusing on maternal and reproductive health–with ANC as a vital component–continues [51].
