# Supplementary material for: Patterns and determinants of antenatal care utilization: analysis of national survey data in seven countdown countries
Source: J Glob Health. 2016 Feb 25;6(1):010404. doi: 10.7189/jogh.06.010404 (PMC4871063; doi:10.7189/jogh.06.010404)
Supplement: Online Supplementary Document [file jogh-06-010404-s001.pdf]

Online Supplementary Document

Saad–Haddad et al. Patterns and determinants of antenatal care utilization: analysis of national survey data in seven countdown countries

JoGH 2016;6:010404

Online Supplementary Document

Table S1: List of 53 Countdown countries (compiled in 2013) that have an accessible DHS survey and their corresponding region, ANC 1+, ANC 4+, priority ranking for eliminating mother-to-child HIV. Countries highlighted in blue were selected for our ANC secondary analysis

	Country	Latest survey year	Region[a]	ANC 1+ coverage[b]	ANC 4+ coverage[c]	SBA coverage[c]	Priority countries for eliminating mother-to-child HIV
1	Azerbaijan	2006	Asia	98.3%	45%	88%	
2	Bangladesh	2011	Asia	51.2%	26%	32%	
3	Cambodia	2010	Asia	89.0%	59%	71%	
4	India	2005	Asia	75.2%	37%	52%	Yes
5	Indonesia	2007	Asia	93.3%	82%	79%	
6	Kyrgyzstan	1997	Asia	96.9%	81%[c]	99%	
7	Myanmar	2000	Asia	79.8%	73%[c]	71%	
8	Nepal	2011	Asia	58.0%	50%	36%	
9	Pakistan	2006	Asia	60.9%	28%	43%	
10	Philippines	2008	Asia	91.1%	78%	62%	
11	Uzbekistan	1996	Asia	99.0%	79%[c]	100%	
12	Vietnam	2002	Asia	90.8%	60%	93%	
13	Guatemala	1998	Central America	93.2%	na	52%	
14	Burundi	2010	Eastern Africa	98.9%	33%	60%	Yes
15	Comoros	1996	Eastern Africa	75.0%	52%[c]	62%	
16	Ethiopia	2011	Eastern Africa	34.0%	19%	10%	Yes
17	Kenya	2008	Eastern Africa	91.5%	47%	44%	Yes
18	Madagascar	2008	Eastern Africa	86.3%	49%	44%	
19	Malawi	2010	Eastern Africa	95.0%	46%	71%	Yes
20	Mozambique	2003	Eastern Africa	92.3%	53%[c]	55%	Yes
21	Rwanda	2010	Eastern Africa	98.0%	35%	69%	
22	Tanzania	2010	Eastern Africa	87.8%	43%	49%	Yes
23	Uganda	2011	Eastern Africa	93.5%	47%	57%	Yes
24	Zambia	2007	Eastern Africa	93.7%	60%	47%	Yes

25	Zimbabwe	2010	Eastern Africa	89.8%	65%	66%	Yes
26	Cameroon	2011	Middle Africa	85.0%	60%[d]	64%	Yes
27	Central African Republic	1994	Middle Africa	69.3%	38%	54%	
28	Chad	2004	Middle Africa	53.1%	23%	23%	
29	Congo_(Brazzaville)	2005	Middle Africa	85.8%	75%[d]	83%	
30	Congo_Democratic_Republic	2007	Middle Africa	87.7%	45%	80%	Yes
31	Gabon	2000	Middle Africa	94.4%	63%	86%	
32	Sao_Tome_and_Principe	2008	Middle Africa	97.9%	72%	82%	
33	Egypt	2008	Northern Africa	73.8%	66%	79%	
34	Morocco	2003	Northern Africa	67.8%	31%[d]	74%	
35	Bolivia	2008	South America	85.8%	72%	71%	
36	Brazil	1996	South America	98.2%	91%	97%	
37	Peru	2012	South America	94.7%	94%	85%	
38	Lesotho	2009	Southern Africa	91.8%	70%	62%	Yes
39	South_Africa	1998	Southern Africa	97.1%	87%	91%	Yes
40	Swaziland	2006	Southern Africa	97.0%	77%	82%	Yes
41	Haiti	2005	The Carribean	84.5%	54%	26%	
42	Benin	2006	Western Africa	84.1%	61%	84%	
43	Burkina_Faso	2010	Western Africa	85.0%	18%[d]	66%	
44	Cote_dIvoire	1998	Western Africa	84.8%	na	59%	Yes
45	Ghana	2008	Western Africa	90.1%	87%	68%	Yes
46	Guinea	2005	Western Africa	88.4%	50%	46%	
47	Liberia	2007	Western Africa	79.3%	66%	46%	
48	Mali	2006	Western Africa	70.4%	35%	49%	
49	Niger	2006	Western Africa	46.1%	15%	18%	
50	Nigeria	2008	Western Africa	57.7%	45%	39%	Yes

51	Senegal	2010	Western Africa	93.0%	50%	65%	
52	Sierra_Leone	2008	Western Africa	86.9%	75%	63%	
53	Togo	1998	Western Africa	86.7%	55%	59%	

ANC – Antenatal care, DHS – Demographic and Health Survey.
The following 22 Countdown countries were excluded from the data selection process because they did not have a DHS Survey: Afghanistan, Angola, Botswana, China, Djibouti, Equatorial Guinea, Eritria, Gambia, Guinea-Bissau, Iraq, Korea (Democratic People's Republic of), Lao People's Democratic Republic, Mauritania, Mexico, Papua New Guinea, Solomon Islands, Somalia, South Sudan, Sudan, Turkmenistan, Tajikistan, Yemen
Notes:
[a] World regions are based on United Nations country grouping. Retrieved from: http://www.internetworldstats.com/list1.htm
[b] Source: maternal and newborn coverage dataset with UNICEF estimates of ANC 1+ for the Countdown countries - updated April 2012.
[c] Source: Countdown to 2015 Report: Accountability for Maternal, Newborn & Child Survival -2013 Report
[d] Source: Countdown to 2015 Report: Building a Future for Women and Children - The 2012 Report

Table S2: ANC providers categorized by type of provider based on the Demographic and Health Survey and Countdown classification, in seven Countdown countries

Provider type / DHS Survey	Skilled provider	Unskilled provider
**Bangladesh 2011**	Doctor, nurse, midwife, paramedic, family welfare visitor, skilled birth attendant, medical assistant (MA) & sub-assistant community medical officer (SACMO)	Health assistant, traditional birth attendant, unqualified doctor, untrained traditional birth attendant, family welfare assistant, non-governmental organization worker & any other provider
**Cambodia 2010**	Doctor, medical assistant, midwife & nurse	Traditional birth attendant, village health volunteer & any other provider
**Cameroon 2011**	Doctor, nurse, midwife & auxiliary midwife	Traditional birth attendant, community health worker & any other provider
**Nepal 2011**	Doctor, nurse & midwife	Health assistant, auxiliary health worker, maternal and child health worker, village health worker, traditional birth attendant, family community health volunteer & any other provider
**Peru 2012**	Doctor, obstetrician, nurse & midwife	Health specialist (technical nurse), health worker, trained birth attendant, traditional birth attendant, relative & any other provider
**Senegal 2010**	Doctor, nurse & midwife	Trained birth attendant, traditional birth attendant & any other provider

**Uganda 2011**	Doctor, nurse, midwife, medical assistant & clinical officer.	Traditional birth attendant, community/village health team & any other provider

Table S3: Percentage of women receiving content interventions during any ANC visit by type of provider for their last live birth in the five years preceding the DHS survey, in seven Countdown countries.

Country	Provider type	Reported 1-3 ANC visits				Reported 4 or more ANC visits			
		blood pressure taken	Urine sample taken	Blood sample taken	Told about pregnancy complications	blood pressure taken	Urine sample taken	Blood sample taken	Told about pregnancy complications
Bangladesh	Skilled				62.2%				75.7%
	Unskilled				54.6%				73.4%
Cambodia	Skilled	82.1%	24.0%	29.4%	73.3%	95.1%	42.7%	52.2%	74.0%
	Unskilled	47.7%	17.6%	6.2%	49.5%	76.4%	15.8%	48.6%	57.1%
Cameroon	Skilled	92.0%	80.4%	81.2%	41.5%	96.8%	90.9%	91.8%	52.8%
	Unskilled	42.2%	31.0%	31.9%	33.9%	77.9%	50.7%	56.5%	49.3%
Nepal	Skilled	89.2%	57.8%	44.0%	72.4%	96.6%	78.6%	69.4%	89.1%
	Unskilled	13.2%	58.8%	5.0%	44.7%	86.5%	32.6%	18.1%	77.7%
Peru	Skilled	98.1%	67.4%	65.4%	78.0%	99.3%	93.0%	93.9%	93.4%
	Unskilled	94.6%	30.7%	31.9%	54.8%	98.6%	60.5%	60.5%	89.1%
Senegal	Skilled	97.0%	79.8%	68.3%	41.5%	98.2%	89.9%	83.8%	48.4%
	Unskilled	84.6%	58.1%	41.2%	38.5%	91.2%	76.4%	60.2%	50.6%
Uganda	Skilled	50.6%	16.1%	77.9%	46.3%	67.4%	27.9%	63.6%	55.9%
	Unskilled	10.6%	0.0%	28.0%	25.1%	47.9%	29.1%	56.9%	38.4%

ANC – antenatal care. DHS – Demographic and health surveys

Note: In Bangladesh, data on the first three interventions: blood pressure taken, urine sample taken and blood sample taken, were not collected in the DHS survey.

Table S4: Percentage of women receiving content interventions during any ANC visit by household wealth quintile among women reporting one to three ANC visits or four or more ANC visits for their last live birth in the five years preceding the DHS survey, in seven Countdown countries.

Country	Wealth quintile	Reported 1-3 ANC visits				Reported 4 or more ANC visits			
		blood pressure taken	Urine sample taken	Blood sample taken	Told about pregnancy complications	blood pressure taken	Urine sample taken	Blood sample taken	Told about pregnancy complications
Bangladesh	Poorest				59.7%				68.1%
	Poorer				57.3%				69.0%
	Middle				63.9%				80.0%
	Richer				59.0%				73.0%
	Richest				62.9%				78.0%
Cambodia	Poorest	79.6%	20.5%	27.4%	68.7%	93.1%	35.9%	43.5%	79.8%
	Poorer	79.6%	20.5%	23.2%	68.9%	94.3%	31.8%	41.3%	83.3%
	Middle	82.5%	23.1%	31.6%	73.1%	94.9%	35.4%	45.8%	83.9%
	Richer	83.1%	31.2%	31.8%	75.3%	94.7%	41.9%	49.2%	83.6%
	Richest	89.7%	34.6%	40.5%	78.8%	97.5%	61.8%	79.5%	87.2%
Cameroon	Poorest	83.3%	60.5%	62.2%	37.1%	83.7%	57.6%	65.6%	40.0%
	Poorer	92.6%	77.9%	80.2%	38.3%	94.8%	86.7%	87.5%	50.9%
	Middle	93.3%	87.3%	87.9%	42.8%	98.3%	94.6%	94.1%	55.2%
	Richer	94.5%	90.8%	88.9%	43.1%	99.4%	97.8%	97.7%	53.4%
	Richest	96.3%	91.3%	90.9%	48.4%	99.8%	98.3%	98.6%	55.7%
Nepal	Poorest	70.1%	23.8%	12.5%	61.1%	86.5%	41.3%	30.9%	81.3%
	Poorer	72.8%	26.3%	17.3%	57.3%	90.7%	50.3%	35.8%	85.3%
	Middle	74.6%	37.4%	29.7%	53.7%	94.9%	61.9%	49.1%	83.6%
	Richer	80.9%	62.8%	44.8%	60.6%	96.6%	81.7%	71.1%	90.2%
	Richest	88.9%	68.9%	52.2%	78.8%	99.0%	90.2%	85.2%	89.9%
Peru	Poorest	95.0%	48.0%	50.3%	77.1%	99.1%	80.8%	82.1%	90.5%
	Poorer	100.0%	67.1%	65.4%	88.0%	99.2%	91.8%	92.4%	94.6%
	Middle	100.0%	85.3%	84.4%	71.3%	99.1%	95.4%	96.2%	94.2%
	Richer	98.7%	89.8%	73.8%	77.8%	100.0%	97.0%	98.5%	92.5%
	Richest	100.0%	45.7%	45.7%	35.0%	99.3%	97.3%	98.1%	94.5%
Senegal	Poorest	93.8%	64.6%	53.0%	37.9%	95.1%	74.0%	69.4%	42.6%
	Poorer	96.5%	77.1%	65.8%	40.4%	96.7%	87.6%	78.4%	48.9%
	Middle	97.2%	86.6%	70.4%	40.6%	97.8%	91.7%	81.7%	47.3%
	Richer	98.3%	90.2%	77.6%	45.4%	99.4%	93.2%	89.6%	47.3%
	Richest	99.6%	90.1%	86.0%	47.7%	99.5%	94.6%	90.1%	53.0%
Uganda	Poorest	50.4%	15.2%	72.0%	49.0%	64.6%	16.8%	80.8%	57.4%
	Poorer	42.8%	12.4%	76.4%	44.9%	55.5%	21.6%	77.4%	54.7%
	Middle	43.7%	13.2%	77.2%	42.4%	59.3%	21.3%	79.4%	49.8%
	Richer	51.0%	16.4%	77.5%	44.3%	64.8%	25.9%	83.8%	48.4%
	Richest	68.7%	25.3%	86.7%	49.0%	85.1%	47.0%	92.1%	63.8%

ANC – antenatal care. DHS – Demographic and health surveys

Figure S5: Percentage of women who had a live birth in the five years preceding the DHS surveys by gestational age at their first ANC visit for their most recent birth, in six Countdown countries.

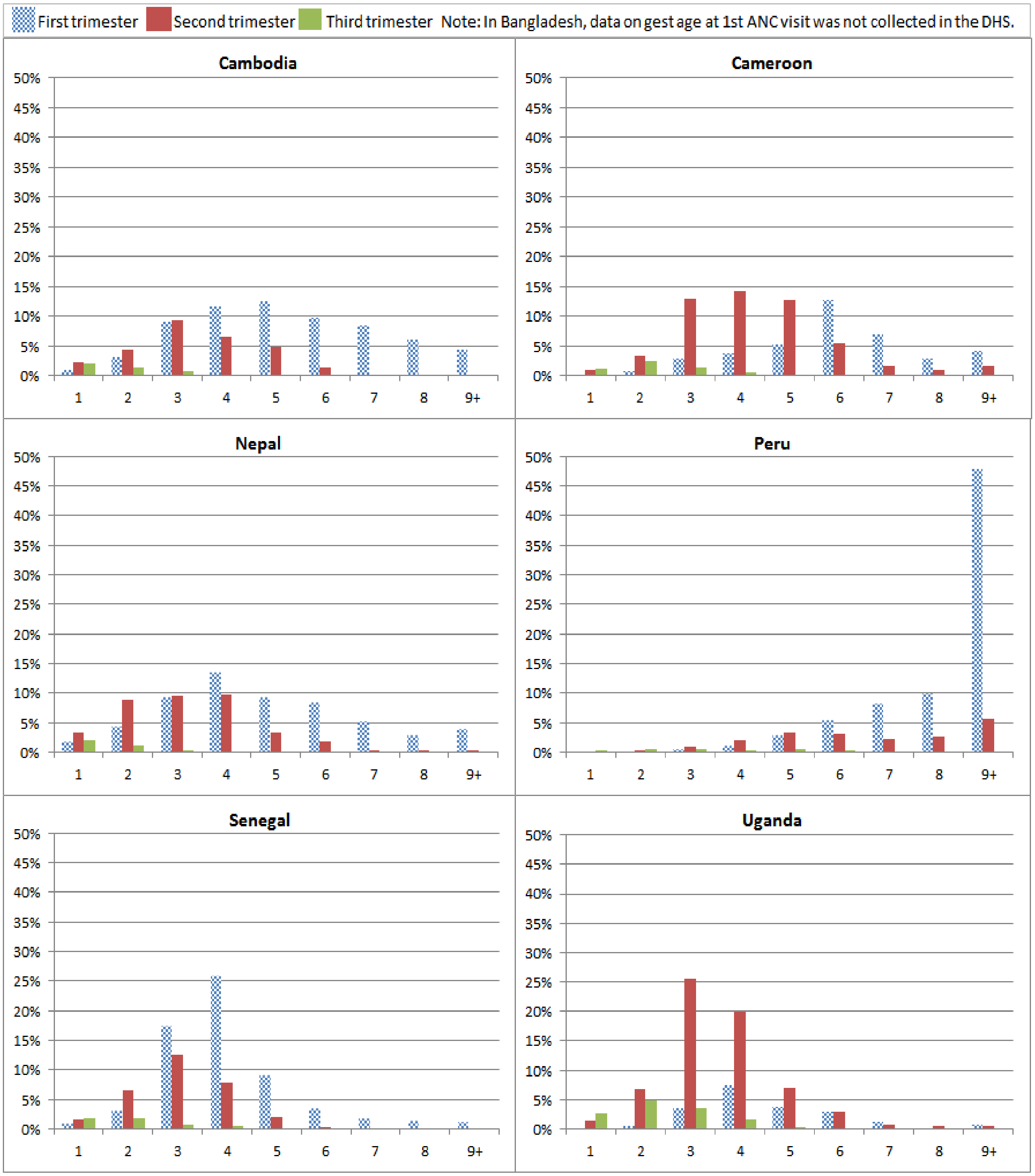

ANC – antenatal care. DHS – Demographic and health surveys

Figure S6.A: Percentage of women who had a live birth in the five years preceding the DHS surveys by number of ANC visits and women's educational level, in seven Countdown countries.

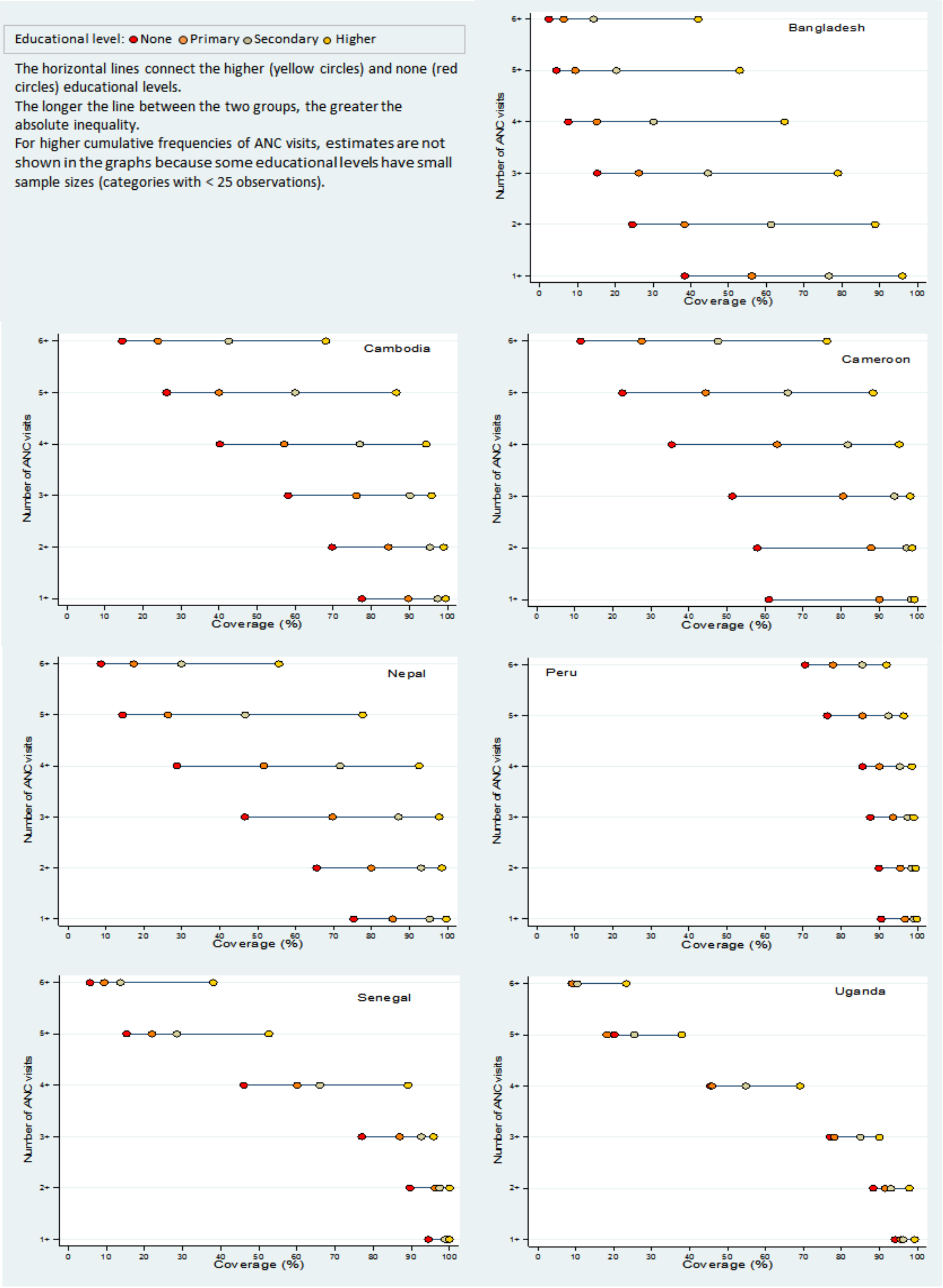

ANC – antenatal care. DHS – Demographic and health surveys

Figure S6.B: Percentage of women who had a live birth in the five years preceding the DHS surveys by number of ANC visits and place of residence, in seven Countdown countries.

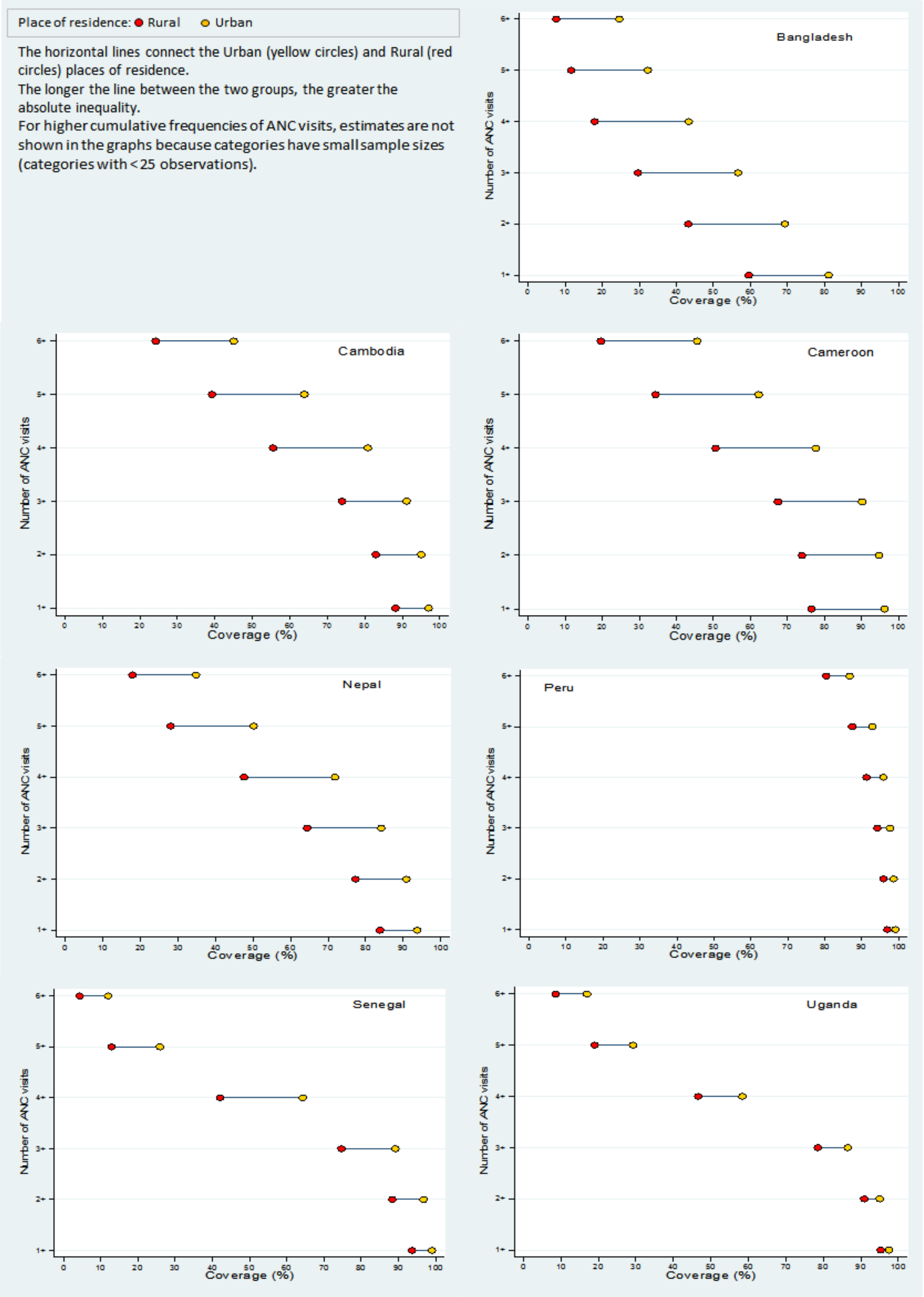

ANC – antenatal care. DHS – Demographic and health surveys

Table S7.A: Multivariable analysis results for Bangladesh after hierarchical enter of the determinants into the analysis in Model A (No ANC visits versus one or more ANC visits) and Model B (one to three ANC visits versus four or more ANC visits)

		**Final Model A (0 visits vs. 1+)**			**Final Model B (1-3 vs. 4+)**		
**Determinants**	**Description**	**OR**	**95% CI**	**P-value**	**OR**	**95% CI**	**P-value**
**External Environment**							
Region of the country	Barisal	*Reference*			*Reference*		
	Chittagong	0.72	0.54-0.97	0.032	0.51	0.36-0.73	0.000
	Dhaka	0.82	0.61-1.10	0.180	0.65	0.47-0.91	0.013
	Khulna	1.12	0.83-1.52	0.468	0.82	0.59-1.15	0.247
	Rajshahi	1.41	1.00-2.00	0.052	0.74	0.52-1.06	0.098
	Rangpur	2.02	1.43-2.87	0.000	1.86	1.28-2.72	0.001
	Sylhet	0.63	0.46-0.86	0.004	0.55	0.37-0.81	0.003
Place of residence	Urban	*Reference*			*Reference*		
	Rural	0.67	0.54-0.82	0.000	0.52	0.42-0.65	0.000
**Predisposing characteristics**							
Woman's educational level	No education	*Reference*			*Reference*		
	Primary	1.61	1.33-1.95	0.000	1.58	1.19-2.10	0.002
	Secondary	2.50	2.00-3.12	0.000	2.19	1.67-2.87	0.000
	Higher	7.99	4.47-14.30	0.000	4.23	2.91-6.13	0.000
Religion	Dominant religion				Ref		
	Other religions				1.25	0.97-1.61	0.090
**Enabling factors**							
Partner's educational level	No education	*Reference*			*Reference*		
	Primary	1.11	0.94-1.32	0.221	1.09	0.87-1.38	0.445
	Secondary	1.36	1.10-1.67	0.004	1.37	1.09-1.73	0.008
	Higher	1.79	1.30-2.46	0.000	1.95	1.47-2.59	0.000
Household wealth quintile	Poorest	*Reference*			*Reference*		
	Poorer	0.95	0.79-1.15	0.607	1.16	0.87-1.54	0.325
	Middle	1.37	1.14-1.65	0.001	1.55	1.18-2.02	0.002
	Richer	2.16	1.74-2.70	0.000	2.15	1.63-2.83	0.000
	Richest	4.63	3.47-6.18	0.000	4.08	2.97-5.60	0.000
**Need factors**							
Birth rank	1st-2nd births	*Reference*			*Reference*		
	3rd-4th births	0.96	0.80-1.14	0.627	0.87	0.72-1.04	0.128
	5th births or more	0.75	0.59-0.94	0.012	0.43	0.29-0.64	0.000
Preceding birth interval	First birth	*Reference*			*Reference*		
	Less than 2 years	0.58	0.44-0.75	0.000	0.71	0.51-0.97	0.033
	2-3 years	0.50	0.40-0.61	0.000	0.62	0.48-0.81	0.000
	More than 3 years	0.69	0.57-0.84	0.000	0.80	0.68-0.95	0.010

ANC - antenatal care, OR - odds ratio, 95% CI - 95% confidence interval
Grey cells represent the determinants excluded by backward elimination during hierarchical entry at the level or due to insignificance at the bivariate level.

Table S7.B: Multivariable analysis results for Cambodia after hierarchical enter of the determinants into the analysis in Model A (No ANC visits versus one or more ANC visits) and Model B (one to three ANC visits versus four or more ANC visits)

		Final Model A (0 visits vs. 1+)			Final Model B (1-3 vs. 4+)		
**Characteristics**	**Description**	**OR**	**95% CI**	**P-value**	**OR**	**95% CI**	**P-value**
**External Environment**							
Region of the country	banteay mean chey	*Reference*			*Reference*		
	kampong cham	1.12	0.51-2.46	0.771	0.77	0.43-1.38	0.382
	kampong chhnang	2.02	1.02-3.97	0.043	1.08	0.60-1.96	0.800
	kampong speu	1.38	0.73-2.61	0.326	0.95	0.55-1.64	0.846
	kampong thom	1.14	0.52-2.51	0.750	0.48	0.25-0.91	0.025
	kandal	0.81	0.42-1.56	0.523	0.72	0.40-1.29	0.268
	kratie	0.30	0.16-0.56	0.000	0.27	0.15-0.47	0.000
	phnom penh	2.57	0.73-8.98	0.140	0.84	0.46-1.53	0.573
	prey veng	1.58	0.84-2.96	0.158	0.49	0.27-0.87	0.015
	pursat	2.18	0.73-6.54	0.165	0.70	0.37-1.33	0.276
	siem reap	3.06	1.13-8.24	0.027	1.95	1.05-3.64	0.035
	svay rieng	2.14	0.97-4.72	0.059	0.63	0.36-1.08	0.093
	takeo	5.75	2.60-12.74	0.000	0.60	0.34-1.05	0.073
	otdar mean chey	1.83	0.73-4.57	0.198	0.81	0.44-1.52	0.515
	battambang & pailin	1.61	0.76-3.42	0.213	1.32	0.72-2.42	0.369
	kampot & kep	0.88	0.44-1.75	0.716	0.36	0.20-0.63	0.000
	preah sihanouk & kaoh kong	0.89	0.38-2.04	0.776	0.53	0.28-0.97	0.041
	preah vihear & steung treng	0.40	0.19-0.82	0.013	0.48	0.24-0.96	0.039
	mondol kiri & rattanak kiri	0.36	0.18-0.72	0.004	0.46	0.26-0.80	0.007
Place of residence	Urban	*Reference*			*Reference*		
	Rural	0.83	0.51-1.33	0.437	0.75	0.55-1.02	0.068
**Predisposing characteristics**							
Woman's age at last birth	<20	*Reference*			*Reference*		
	20-34	1.58	1.00-2.50	0.051	1.43	1.07-1.91	0.017
	35-49	1.35	0.78-2.36	0.284	1.48	0.98-2.24	0.064
Woman's educational level	No education	*Reference*			*Reference*		
	Primary	1.56	1.20-2.05	0.001	1.27	1.00-1.61	0.046
	Secondary	2.80	1.67-4.69	0.000	1.62	1.21-2.16	0.001
	Higher	5.98	0.60-59.42	0.126	2.94	0.84-10.25	0.090
Woman's employment status	Did not work in the past 12 months				*Reference*		
	Worked in the past 12 months				0.88	0.70-1.11	0.286
Religion	Dominant religion				*Reference*		
	Other religions				0.51	0.30-0.86	0.012
**Enabling factors**							
Partner's educational level	No education	*Reference*			*Reference*		
	Primary	1.13	0.85-1.51	0.405	1.16	0.88-1.53	0.283
	Secondary	1.86	1.33-2.59	0.000	1.59	1.16-2.18	0.004

	Higher	1.19	0.32-4.47	0.795	2.13	1.17-3.87	0.014
Household wealth quintile	Poorest	*Reference*			*Reference*		
	Poorer	1.22	0.94-1.60	0.140	1.23	0.97-1.55	0.089
	Middle	1.97	1.44-2.70	0.000	1.07	0.81-1.40	0.633
	Richer	2.83	1.87-4.28	0.000	1.59	1.19-2.12	0.002
	Richest	4.60	2.36-8.97	0.000	1.71	1.20-2.44	0.003
**Need factors**							
Gestational age at initial visit	First trimester	*Determinant excluded from the analysis in Model A*			*Reference*		
	Second trimester				0.23	0.20-0.27	0.000
	Third trimester				0.02	0.01-0.04	0.000
Birth rank	1st-2nd births	*Reference*			*Reference*		
	3rd-4th births	0.60	0.43-0.83	0.002	0.85	0.69-1.05	0.124
	5th births or more	0.32	0.22-0.47	0.000	0.55	0.40-0.76	0.000
Preceding birth interval	First birth	*Reference*			*Reference*		
	Less than 2 years	0.47	0.32-0.70	0.000	0.75	0.55-1.03	0.076
	2-3 years	0.47	0.31-0.71	0.000	0.82	0.63-1.08	0.156
	More than 3 years	0.65	0.44-0.95	0.025	0.71	0.56-0.91	0.005

ANC - antenatal care, OR - odds ratio, 95% CI - 95% confidence interval
Grey cells represent the determinants excluded by backward elimination during hierarchical entry at the level or due to insignificance at the bivariate level.

Table S7.C: Multivariable analysis results for Cameroon after hierarchical enter of the determinants into the analysis in Model A (No ANC visits versus one or more ANC visits) and Model B (one to three ANC visits versus four or more ANC visits)

		**Final Model A (0 visits vs. 1+)**			**Final Model B (1-3 vs. 4+)**		
**Characteristics**	**Description**	**OR**	**95% CI**	**P-value**	**OR**	**95% CI**	**P-value**
**External Environment**							
Region of the country	Adamaoua	*Reference*			*Reference*		
	Centre	0.56	0.30-1.06	0.075	1.25	0.84-1.85	0.264
	Douala	3.47	0.75-16.18	0.113	2.68	1.64-4.38	0.000
	Est	0.40	0.25-0.65	0.000	0.70	0.49-1.00	0.051
	Extreme-Nord	0.32	0.20-0.50	0.000	1.61	1.14-2.28	0.007
	Littoral	1.93	0.70-5.32	0.203	1.41	0.93-2.15	0.109
	Nord	0.58	0.37-0.92	0.020	2.15	1.48-3.14	0.000
	Nord-ouest	9.41	3.19-27.73	0.000	5.32	3.46-8.19	0.000
	Ouest	5.55	2.19-14.07	0.000	1.83	1.21-2.76	0.004
	Sud	0.57	0.28-1.18	0.132	0.51	0.34-0.76	0.001
	Sud-ouest	0.50	0.25-0.99	0.047	2.10	1.38-3.18	0.001
	Yaoude	0.93	0.38-2.32	0.884	1.70	1.10-2.62	0.016
Place of residence	Urban	*Reference*			*Reference*		
	Rural	0.72	0.48-1.08	0.112	0.92	0.71-1.19	0.529
**Predisposing characteristics**							
Woman's age at last birth	<20				*Reference*		
	20-34				1.57	1.24-1.97	0.000

	35-49				1.46	1.06-2.01	0.021
Woman's educational level	No education	*Reference*			*Reference*		
	Primary	1.51	1.16-1.95	0.002	1.15	0.88-1.50	0.312
	Secondary	3.75	2.39-5.89	0.000	1.56	1.13-2.16	0.007
	Higher	2.18	0.40-11.94	0.368	3.89	1.85-8.16	0.000
Religion	Dominant religion	*Reference*					
	Other religions	0.70	0.52-0.95	0.024			
Marital status	Currently in union	*Reference*			*Reference*		
	Formerly/never in union	0.92	0.54-1.58	0.770	0.84	0.60-1.18	0.318
**Enabling factors**							
Partner's educational level	No education	*Reference*			*Reference*		
	Primary	1.72	1.24-2.39	0.001	1.06	0.82-1.36	0.677
	Secondary	2.45	1.65-3.64	0.000	1.18	0.87-1.59	0.288
	Higher	6.32	1.13-35.27	0.036	1.20	0.73-1.96	0.466
Household wealth quintile	Poorest	*Reference*			*Reference*		
	Poorer	1.60	1.18-2.16	0.002	1.17	0.93-1.46	0.176
	Middle	3.02	1.99-4.58	0.000	1.19	0.89-1.59	0.241
	Richer	3.82	2.02-7.22	0.000	1.60	1.13-2.26	0.008
	Richest	6.07	2.70-13.64	0.000	1.84	1.24-2.73	0.002
Decision re woman's health care	woman alone	*Reference*					
	Woman & partner	0.79	0.53-1.18	0.259			
	Partner alone	0.93	0.62-1.40	0.744			
	someone else/other	0.34	0.14-0.82	0.017			
**Need factors**							
Gestational age at initial visit	First trimester	*Determinant excluded from the analysis in Model A*			*Reference*		
	Second trimester				0.24	0.20-0.28	0.000
	Third trimester				0.03	0.02-0.04	0.000
Preceding birth interval	First birth	*Reference*			*Reference*		
	Less than 2 years	0.67	0.47-0.96	0.030	0.64	0.47-0.86	0.004
	2-3 years	0.60	0.42-0.84	0.003	0.72	0.55-0.95	0.022
	More than 3 years	0.63	0.47-0.85	0.002	0.76	0.59-0.98	0.032

ANC - antenatal care, OR - odds ratio, 95% CI - 95% confidence interval
Grey cells represent the determinants excluded by backward elimination during hierarchical entry at the level or due to insignificance at the bivariate level.

Table S7.D: Multivariable analysis results for Nepal after hierarchical enter of the determinants into the analysis in Model A (No ANC visits versus one or more ANC visits) and Model B (one to three ANC visits versus four or more ANC visits)

		Final Model A (0 visits vs. 1+)			Final Model B (1-3 vs. 4+)		
Characteristics	Description	OR	95% CI	P-value	OR	95% CI	P-value
**External Environment**							
Region of the country	Mountain	*Reference*					
	Hill	0.77	0.49-1.21	0.258			
	Terai	1.51	0.92-2.46	0.102			
Place of residence	Urban	*Reference*			*Reference*		
	Rural	1.08	0.70-1.69	0.735	0.88	0.64-1.22	0.454
**Predisposing characteristics**							
Woman's age at last birth	<20	*Reference*			*Reference*		
	20-34	1.54	1.05-2.25	0.026	1.17	0.88-1.56	0.278
	35-49	0.99	0.57-1.72	0.966	1.21	0.70-2.10	0.490
Woman's educational level	No education	*Reference*			*Reference*		
	Primary	1.17	0.82-1.67	0.375	1.75	1.32-2.32	0.000
	Secondary	1.99	1.22-3.26	0.006	2.28	1.72-3.01	0.000
	Higher	14.39	1.73-119.80	0.014	6.12	2.57-14.58	0.000
**Enabling factors**							
Partner's educational level	No education	*Reference*			*Reference*		
	Primary	1.32	0.95-1.84	0.097	1.44	1.05-1.98	0.024
	Secondary	1.66	1.17-2.35	0.005	1.56	1.12-2.18	0.009
	Higher	1.97	0.91-4.26	0.083	1.28	0.81-2.03	0.292
Household wealth quintile	Poorest	*Reference*			*Reference*		
	Poorer	1.40	1.03-1.91	0.032	1.04	0.77-1.41	0.795
	Middle	2.22	1.32-3.71	0.003	1.05	0.72-1.54	0.800
	Richer	2.60	1.52-4.44	0.001	1.55	1.04-2.33	0.033
	Richest	5.55	2.39-12.85	0.000	2.36	1.44-3.85	0.001
Decision re woman's health care	woman alone	*Reference*			*Reference*		
	Woman & partner	1.05	0.77-1.44	0.755	0.93	0.72-1.19	0.554
	Partner alone	0.64	0.45-0.91	0.014	0.72	0.53-0.99	0.041
	someone else	0.72	0.46-1.13	0.153	0.69	0.52-0.91	0.010
**Need factors**							
Gestational age at initial visit	First trimester	*Determinant excluded from the analysis in Model A*			*Reference*		
	Second trimester				0.36	0.29-0.46	0.000
	Third trimester				0.02	0.01-0.06	0.000
Birth rank	1st-2nd births	*Reference*			*Reference*		
	3rd-4th births	0.71	0.54-0.92	0.009	0.85	0.65-1.11	0.223
	5th births or more	0.41	0.29-0.59	0.000	0.58	0.37-0.91	0.018
Preceding birth interval	First birth	*Reference*			*Reference*		
	Less than 2 years	0.59	0.36-0.94	0.028	0.60	0.42-0.86	0.005
	2-3 years	0.58	0.39-0.86	0.007	0.69	0.51-0.95	0.021
	More than 3 years	0.54	0.35-0.83	0.005	0.84	0.65-1.10	0.215

ANC - antenatal care, OR - odds ratio, 95% CI - 95% confidence interval
Grey cells represent the determinants excluded by backward elimination during hierarchical entry at the level or due to insignificance at the bivariate level.

Table S7.E: Multivariable analysis results for Peru after hierarchical enter of the determinants into the analysis in Model A (No ANC visits versus one or more ANC visits) and Model B (one to three ANC visits versus four or more ANC visits)

		Final Model A (0 visits vs. 1+)			Final Model B (1-3 vs. 4+)		
**Characteristics**	**Description**	**OR**	**95% CI**	**P-value**	**OR**	**95% CI**	**P-value**
**External Environment**							
Region of the country	Amazonas	*Reference*			*Reference*		
	Ancash	4.72	1.02-21.91	0.047	1.56	0.64-3.80	0.325
	Apurimac	10.49	2.26-48.72	0.003	2.14	0.90-5.07	0.085
	Arequipa	4.92	0.73-33.07	0.101	2.12	0.67-6.73	0.203
	Ayacucho	16.70	3.21-86.96	0.001	2.06	0.92-4.60	0.078
	Cajamarca	5.43	1.85-15.93	0.002	2.30	0.89-5.98	0.086
	Callao	1.00			2.07	0.43-9.95	0.365
	Cusco	21.44	2.48-185.15	0.005	1.37	0.58-3.26	0.470
	Huancavelica	7.93	2.28-27.60	0.001	2.71	1.16-6.33	0.021
	Huanuco	50.15	5.73-439.22	0.000	2.83	1.27-6.33	0.011
	Ica	2.88	0.47-17.46	0.250	0.87	0.36-2.12	0.757
	Junin	4.83	1.24-18.88	0.023	0.83	0.39-1.78	0.637
	La Libertad	3.74	1.33-10.52	0.012	1.25	0.59-2.65	0.564
	Lambayeque	2.20	0.66-7.26	0.197	1.16	0.51-2.65	0.727
	Lima	3.48	0.68-17.86	0.134	1.50	0.59-3.78	0.392
	Loreto	1.18	0.49-2.85	0.705	0.84	0.41-1.74	0.640
	Madre de Dios	3.04	0.92-10.11	0.069	0.74	0.33-1.67	0.469
	Moquegua	2.98	0.30-29.90	0.352	2.49	0.83-7.51	0.105
	Pasco	3.31	0.97-11.32	0.056	2.13	0.87-5.18	0.097
	Piura	4.30	1.34-13.83	0.015	1.45	0.62-3.37	0.387
	Puno	5.02	1.62-15.52	0.005	1.33	0.64-2.74	0.447
	San Martin	2.32	0.90-6.01	0.082	1.84	0.76-4.47	0.178
	Tacna	1.72	0.30-9.92	0.545	2.01	0.51-7.95	0.321
	Tumbes	0.95	0.29-3.16	0.934	0.71	0.31-1.63	0.422
	Ucayali	0.86	0.31-2.40	0.778	0.43	0.21-0.86	0.017
Place of residence	Urban	*Reference*			*Reference*		
	Rural	0.83	0.48-1.43	0.499	1.05	0.70-1.58	0.814
**Predisposing characteristics**							
Woman's age at last birth	<20				*Reference*		
	20-34				2.48	1.46-4.20	0.001
	35-49				1.67	0.82-3.37	0.155
Woman's educational level	No education	*Reference*			*Reference*		
	Primary	2.44	1.28-4.67	0.007	0.46	0.21-1.03	0.058
	Secondary	3.88	1.64-9.19	0.002	0.74	0.31-1.79	0.508
	Higher	10.52	3.26-33.98	0.000	0.95	0.32-2.78	0.923
Marital status	Currently in union	*Reference*			*Reference*		
	Formerly/never in union	0.74	0.25-2.15	0.577	1.29	0.49-3.36	0.607
**Enabling factors**							

Partner's educational level	No education	*Reference*			*Reference*		
	Primary	2.28	0.81-6.46	0.120	3.20	1.39-7.37	0.006
	Secondary	2.64	0.92-7.58	0.070	2.48	1.04-5.92	0.041
	Higher	6.77	0.73-62.95	0.093	2.10	0.67-6.62	0.205
Household wealth quintile	Poorest	*Reference*			*Reference*		
	Poorer	1.44	0.85-2.45	0.177	1.36	0.92-2.02	0.122
	Middle	2.30	0.92-5.74	0.074	1.41	0.75-2.66	0.282
	Richer	6.83	2.00-23.28	0.002	1.29	0.58-2.85	0.529
	Richest	1.00			3.07	0.82-11.49	0.095
Decision re woman's health care	woman alone				*Reference*		
	Woman & partner				0.81	0.53-1.25	0.347
	Partner alone				0.65	0.42-0.99	0.047
	Someone else/other				0.33	0.06-1.82	0.200
**Need factors**							
Gestational age at initial visit	First trimester	*Determinant excluded from the analysis in Model A*			*Reference*		
	Second trimester				0.18	0.12-027	0.000
	Third trimester				0.01	0.01-0.02	0.000
Birth rank	1st-2nd births	*Reference*			*Reference*		
	3rd-4th births	0.68	0.41-1.13	0.137	0.72	0.45-1.14	0.156
	5th births or more	0.35	0.21-0.59	0.000	0.53	0.30-0.93	0.026

ANC - antenatal care, OR - odds ratio, 95% CI - 95% confidence interval
Grey cells represent the determinants excluded by backward elimination during hierarchical entry at the level or due to insignificance at the bivariate level.

Table s7.F: Multivariable analysis results for Senegal after hierarchical enter of the determinants into the analysis in Model A (No ANC visits versus one or more ANC visits) and Model B (one to three ANC visits versus four or more ANC visits)

		**Final Model A (0 visits vs. 1+)**			**Final Model B (1-3 vs. 4+)**		
**Characteristics**	**Description**	**OR**	**95% CI**	**P-value**	**OR**	**95% CI**	**P-value**
**External Environment**							
Region of the country	Dakar	*Reference*			*Reference*		
	Ziguinchor	2.27	0.36-14.39	0.382	1.10	0.76-1.60	0.600
	Diourbel	0.44	0.14-1.38	0.159	1.09	0.79-1.51	0.588
	Saint-louis	0.32	0.10-1.07	0.065	1.01	0.71-1.43	0.966
	Tambacounda	0.25	0.08-0.74	0.012	0.93	0.62-1.40	0.739
	Kaolack	1.21	0.35-4.26	0.762	0.84	0.61-1.17	0.301
	Thiès	2.18	0.54-8.74	0.271	1.25	0.88-1.76	0.208
	Louga	0.51	0.16-1.57	0.238	1.60	1.10-2.33	0.014
	Fatick	1.06	0.27-4.13	0.928	1.06	0.72-1.56	0.756
	Kolda	0.38	0.12-1.20	0.099	0.48	0.33-0.71	0.000
	Matam	0.18	0.06-0.56	0.003	0.75	0.53-1.07	0.114
	Kaffrine	0.35	0.11-1.10	0.072	0.84	0.54-1.31	0.450
	Kedougou	0.29	0.09-0.99	0.049	1.23	0.74-2.05	0.428
	Sedhiou	0.72	0.21-2.52	0.606	0.80	0.53-1.21	0.296
Place of	Urban	*Reference*			*Reference*		

residence	Rural	0.62	0.39-0.99	0.047	0.74	0.61-0.88	0.001
**Predisposing characteristics**							
Woman's educational level	No education	*Reference*			*Reference*		
	Primary	3.16	1.90-5.25	0.000	1.00	0.83-1.21	0.971
	Secondary	1.02	0.42-2.49	0.960	0.84	0.62-1.14	0.265
	Higher	1.00			1.94	0.58-6.52	0.281
**Enabling factors**							
Partner's educational level	No education				*Reference*		
	Primary				1.11	0.89-1.38	0.340
	Secondary				1.23	0.91-1.67	0.176
	Higher				1.85	1.16-2.94	0.010
Household wealth quintile	Poorest	*Reference*			*Reference*		
	Poorer	1.98	1.42-2.76	0.000	1.17	0.99-1.38	0.061
	Middle	2.60	1.65-4.12	0.000	1.42	1.16-1.73	0.001
	Richer	5.57	2.08-14.91	0.001	1.57	1.20-2.06	0.001
	Richest	7.74	2.40-25.00	0.001	1.95	1.42-2.68	0.000
**Need factors**							
Gestational age at initial visit	First trimester	*Determinant excluded from the analysis in Model A*			*Reference*		
	Second trimester				0.29	0.25-0.35	0.000
	Third trimester				0.08	0.04-0.15	0.000
Birth rank	1st-2nd births	*Reference*			*Reference*		
	3rd-4th births	0.79	0.54-1.13	0.195	0.87	0.73-1.04	0.121
	5th births or more	0.64	0.44-0.95	0.027	0.77	0.65-0.91	0.003
Preceding birth interval	First birth	*Reference*					
	Less than 2 years	0.56	0.33-0.96	0.033			
	2-3 years	0.73	0.44-1.22	0.226			
	More than 3 years	0.75	0.44-1.29	0.302			

ANC - antenatal care, OR - odds ratio, 95% CI - 95% confidence interval
Grey cells represent the determinants excluded by backward elimination during hierarchical entry at the level or due to insignificance at the bivariate level.

Table S7.G: Multivariable analysis results for Uganda after hierarchical enter of the determinants into the analysis in Model A (No ANC visits versus one or more ANC visits) and Model B (one to three ANC visits versus four or more ANC visits)

		Final Model A (0 visits vs. 1+)			Final Model B (1-3 vs. 4+)		
**Characteristics**	**Description**	**OR**	**95% CI**	**P-value**	**OR**	**95% CI**	**P-value**
**External Environment**							
Region of the country	Kampala	*Reference*			*Reference*		
	Central 1	0.29	0.11-0.76	0.012	0.68	0.46-1.00	0.050
	Central 2	0.78	0.28-2.15	0.629	0.76	0.52-1.13	0.175
	East Central	0.48	0.18-1.29	0.143	0.69	0.48-1.00	0.051
	Eastern	0.89	0.30-2.65	0.835	0.57	0.38-0.84	0.005
	North	4.91	1.16-20.76	0.031	0.57	0.36-0.89	0.013
	Karamoja	2.20	0.50-9.62	0.296	0.98	0.61-1.57	0.922
	West-Nile	3.42	0.90-13.08	0.072	1.26	0.83-1.91	0.286

	Western	1.11	0.39-3.14	0.849	0.67	0.39-1.13	0.133
	Southwest	3.04	0.92-10.05	0.069	0.54	0.36-0.80	0.002
Place of residence	Urban						
	Rural						
**Predisposing characteristics**							
Woman's age at last birth	<20	*Reference*					
	20-34	1.62	1.03-2.54	0.035			
	35-49	0.91	0.48-1.71	0.762			
Woman's educational level	No education	*Reference*			*Reference*		
	Primary	1.45	0.90-2.32	0.126	1.05	0.82-1.34	0.721
	Secondary	1.59	0.68-3.71	0.288	1.04	0.77-1.40	0.794
	Higher	6.31	1.08-36.86	0.041	1.46	0.92-2.33	0.106
Religion	Dominant religion	*Reference*					
	Other religions	1.78	1.06-3.00	0.031			
**Enabling factors**							
Partner's educational level	No education						
	Primary						
	Secondary						
	Higher						
Household wealth quintile	Poorest	*Reference*			*Reference*		
	Poorer	1.48	0.86-2.54	0.156	1.16	0.90-1.49	0.244
	Middle	1.62	0.98-2.70	0.062	1.23	0.94-1.61	0.125
	Richer	2.12	1.20-3.72	0.009	1.66	1.21-2.29	0.002
	Richest	2.43	1.16-5.09	0.019	1.98	1.42-2.77	0.000
**Need factors**							
Gestational age at initial visit	First trimester	*Determinant excluded from the analysis in Model A*			*Reference*		
	Second trimester				0.26	0.21-0.32	0.000
	Third trimester				0.05	0.03-0.08	0.000
Pregnancy wanted at the time	Yes	*Reference*					
	Later	0.57	0.37-0.89	0.014			
	No more	0.44	0.25-0.79	0.006			

ANC - antenatal care, OR - odds ratio, 95% CI - 95% confidence interval
Grey cells represent the determinants excluded by backward elimination during hierarchical entry at the level or due to insignificance at the bivariate level.
